# Feature importance network reveals novel functional relationships between biological features in *Arabidopsis thaliana*

**DOI:** 10.3389/fpls.2022.944992

**Published:** 2022-09-23

**Authors:** Jonathan Wei Xiong Ng, Swee Kwang Chua, Marek Mutwil

**Affiliations:** School of Biological Sciences, Nanyang Technological University, Singapore, Singapore

**Keywords:** machine learning, random forest, feature importance, network, *Arabidopsis*, database

## Abstract

Understanding how the different cellular components are working together to form a living cell requires multidisciplinary approaches combining molecular and computational biology. Machine learning shows great potential in life sciences, as it can find novel relationships between biological features. Here, we constructed a dataset of 11,801 gene features for 31,522 *Arabidopsis thaliana* genes and developed a machine learning workflow to identify linked features. The detected linked features are visualised as a Feature Important Network (FIN), which can be mined to reveal a variety of novel biological insights pertaining to gene function. We demonstrate how FIN can be used to generate novel insights into gene function. To make this network easily accessible to the scientific community, we present the FINder database, available at finder.plant.tools.^1^

## Introduction

Recent advances in computational approaches and experimental workflows have made obtaining genome-wide biological and genomic data relatively easy and commonplace. The high-throughput data capture different biological features for DNA (e.g., sequence, methylation, chromatin accessibility, and chromatic conformation) and RNA (e.g., sequence, abundance, structure, and modification) for hundreds of plants (Mahood et al., 2020; Hassani-Pak et al., 2021). However, the sheer volume of biological data presents a challenge for deriving biological meaning from it. As such, identifying how different biological features link to each other, and how they interact with genomic information remains a significant challenge (Rhee and Mutwil, 2014; Greener et al., 2021).

Until recently, the main approach to determine how biological features are linked would use complex statistical approaches that might be sensitive to the quality of the data (Rhee and Mutwil, 2014). Fortunately, machine learning has emerged as a popular technique in many biological contexts, to make predictions based on biological information fed into it. This is because some machine learning algorithms are efficient enough to handle massive data sets that exhibit high amounts of noise, dimensionality, and/or incompleteness, and make minimal assumptions about the data’s underlying probability distributions and generation methods (Mahood et al., 2020). Machine learning methods can usefully be segregated into two primary categories: supervised or unsupervised learning methods. Supervised methods are trained on labelled examples and then used to make predictions about unlabelled examples, whereas unsupervised methods find structure in a data set without using labels (Libbrecht and Noble, 2015; Chang et al., 2021).

Machine learning has been used to predict gene function in multiple contexts, such as predicting specialised metabolism genes (Moore et al., 2019), and computational assignment of GO terms to genes (Zhai et al., 2016; Kulmanov et al., 2018; Zwaenepoel et al., 2018; Kang et al., 2019; Sureyya Rifaioglu et al., 2019; Fu et al., 2020; Littmann et al., 2021). For example, Moore et al. used around 10,000 features on a dataset of around 5,000 genes to predict specialised metabolism genes, achieving a true positive rate of 87% and a true negative rate of 71%. Kulmanov et al. (2018), Sureyya Rifaioglu et al. (2019), and Littmann et al. (2021) used protein sequences as the sole data source for GO term prediction. A recent study (Cheng et al., 2021) used an evolutionarily-informed machine learning approach within and across species to predict genes affecting nitrogen utilisation in crops, and showed how their approach is also useful in mammalian systems.

Machine learning can be also used to infer gene regulatory networks. For example, a study showed that plant metabolism is transcriptionally coordinated *via* developmental and stress conditional processes (Tang et al., 2021). Another approach, named Expression Prediction *via* Log-linear Combination of Transcription Factors (EXPLICIT), correctly predicted gene expression patterns from transcription factor information. EXPLICIT also enabled inference of transcription factor regulators for genes functioning in diverse plant pathways, including those involved in suberin, flavonoid, lateral root, xylem, and the endoplasmic reticulum stress response (Geng et al., 2021). Thus, with the ability to correctly predict gene function and regulation from high-dimensional data, machine learning has a great potential to transform biology.

However, while achieving accurate predictions is a key aim of machine learning, understanding which biological features contribute to making these predictions can reveal how the different biological features are linked. Fortunately, some machine learning approaches are interpretable, as they provide feature importance scores that quantify how important a feature is in predicting the target feature. When five categories of features, gene sequence, protein sequence, network topology, homology, and gene ontology-based features, were used to predict essential genes in *Caenorhabditis elegans*, the topology feature category provided the highest discriminatory power for essentiality prediction (Aromolaran et al., 2021). In using machine learning to predict *Arabidopsis thaliana* secondary metabolism genes, it was shown that multiple genetic features, such as tandem duplication, coexpression with paralogs, expression levels, conservation, and gene coexpression are predictive of secondary metabolism genes relative to general metabolism genes (Moore et al., 2019).

Using reported regulatory pairs in *A. thaliana* along with gene expression and molecular information (Zaborowski and Walther, 2020), the authors of that study aimed to discern the molecular determinants of high expression correlation of transcription factors and their target genes. Specific molecular determinants, such as transcription factor family assignment, stress-response process involvement, and young evolutionary age of target genes were found particularly indicative of high transcription factor target gene correlation.

The above examples showcase the power of machine learning in identifying information that explains the molecular wiring of plants. However, the above-mentioned studies focused on specific aspects of gene function (essentiality, specialised metabolism, and gene regulation), which precludes us from understanding how the different properties of genes are important for their function. In addition, while developing useful machine learning models for such purposes poses a challenge, a second challenge would be to present them in a user-friendly way in which biologists, especially those without a computational background, would be able to access and understand. Such databases exist for many areas of plant biology. Aranet predicts gene function using function gene networks (Lee et al., 2015). SUBA is a database containing experimental and computational predictions of Arabidopsis subcellular protein locations (Hooper et al., 2017). CoNekT-Plants are a database of tissue specific gene expression for seven plant species (Proost and Mutwil, 2018). However, to our knowledge, no databases that portray an extensive machine learning analysis of various *A. thaliana* genetic characteristics exist.

To address this, we constructed an extensive dataset of 31,522 *A. thaliana* genes, drawn from 11,801 features from multiple biological and genomic categories. These features were obtained from a literature search to identity studies where researchers have generated a wide range of computational and experimental data on *A. thaliana*. Examples of such studies include focused on plant secondary metabolism genes (Moore et al., 2019), and the identification of essential plant genes (Lloyd et al., 2015).

*Arabidopsis thaliana* is the plant with most publicly available experimental data. Unfortunately, for non-model species, such a range of experimental data is absent. For example, for Gene Ontology (GO) terms based on experimental evidence, 91,436 *A. thaliana* annotations are present, compared to a few hundred annotations for other plants (obtained from http://amigo.geneontology.org/amigo/search/annotation at 30 May 2022). Hence, other plant species were not used.

We then used a machine learning workflow on this dataset to test the predictability of all features and observed that certain features are more predictable than others. Using feature importance values derived from our workflow, we constructed a Feature Importance Network (FINder), which can be used to study which of the 11,801 features are putatively functionally related. To make our analyses publicly-available, we created an online database, finder.plant.tools.^2^ With FINder, we exemplify how potential novel biological relationships amongst features can be identified.

## Materials and methods

### Sequence information

Primary transcripts of *A. thaliana* coding sequences (CDS) and protein sequences are obtained from Phytozome (http://phytozome.jgi.doe.gov/pz/portal.html; Goodstein et al., 2012).

### Gene expression features

Gene expression levels as measured by transcript per million (TPM) values, and gene specificity measure (SPM) values were obtained from EVOREPRO (www.evorepro.plant.tools; Julca et al., 2021). Differential gene expression (DGE) features were obtained from RNA sequencing (RNA-seq) data from ArrayExpress (https://www.ebi.ac.uk/arrayexpress/; Athar et al., 2019), and processed with kallisto (Bray et al., 2016) and sleuth (Pimentel et al., 2017). Amplitude and time points of peak expression of diurnal genes, was downloaded from diurnal.plant.tools (https://diurnal.sbs.ntu.edu.sg/; Ng et al., 2020).

### Gene family, phylostrata, and genomic information features

Gene families, defined as orthogroups, as well as phylostrata corresponding to each gene, were downloaded from EVOREPRO (Julca et al., 2021). Smaller numbers (starting from 1) indicate older phylostrata and larger numbers (ending at 21) indicate younger phylostrata. Gene family size, single copy, and tandemly duplicated genes were also identified.

### Protein domain and biochemical features

InterProScan 5.44-79.0 (Jones et al., 2014) on *A. thaliana* protein sequences was run, and the number of protein domains (Pfam), disordered regions (MobiDBLite) and transmembrane helices (TMHMM) were obtained. The total number of domains in each gene, protein length, isoelectric point (*pI*), and molecular weight of proteins were obtained, with the last two from the Isoelectric Point Calculator (IPC; Kozlowski, 2016).

### Biological network features

Biological network features were made from Protein protein interaction (PPI; BioGRID, https://thebiogrid.org/; Stark et al., 2006), gene coexpression (EVOREPRO; Julca et al., 2021), gene regulatory (Zaborowski and Walther, 2020), and functional gene networks (Aranet, http://www.inetbio.org/aranet/; Lee et al., 2015). For all networks, two network centrality measures and degree and betweenness centrality were calculated. A markov cluster (MCL) algorithm (Van Dongen, 2000; Enright et al., 2002) was used to cluster the PPI, gene regulatory and functional gene networks. The heuristic cluster chiselling algorithm (HCCA; Mutwil et al., 2010) was used to cluster the gene coexpression network through the calculation of highest reciprocal rank (HRR). HRR defines the mutual coexpression relationship between two genes of interest.

### Experimental GO terms as features

Gene annotations in the form of gene ontology (GO) terms were downloaded from The Arabidopsis Information Resource (TAIR, http://arabidopsis.org; Berardini et al., 2015). Only gene annotations with experimental evidence codes EXP, IDA, IPI, IMP, IGI, and IEP were selected.

### Cis-regulatory element features

Cis-regulatory element names and families were downloaded from the Arabidopsis Gene Regulatory Information Server (AGRIS) database (https://agris-knowledgebase.org/; Yilmaz et al., 2011). Their frequency per gene was calculated.

### Multi-omics (genomic and transcriptomic associated) features

Multi-omics features, in the form of GWAS and transcriptome-wide association studies (TWAS) were downloaded from the *Arabidopsis thaliana* multi-omics association (AtMAD) database (http://119.3.41.228/atmad/index.php; Lan et al., 2021). The number of times each gene was associated with each phenotype trait was counted.

### Evolutionary/conservation and epigenetic features

Homologous features were obtained from the EVOREPRO database (Julca et al., 2021). Nucleotide diversity, methylation status of gene bodies, and sequence conservation features were obtained from a 2015 study (Lloyd et al., 2015).

### Protein post-translational modification features

Protein post-translational modification (PTM) features were obtained from the Plant PTM Viewer (http://www.psb.ugent.be/PlantPTMViewer; Willems et al., 2019). For each gene, the number of each PTM together with the amino acid which it occurs in, was counted.

### Data preprocessing

All features were combined to create a dataset comprising 11,801 features and 31,552 genes for machine learning (Supplementary Data 1). Missing categorical features were filled with 0. Missing continuous features were filled with their respective mean values, and continuous features were standardised (*z*-score normalisation). Features with >50% of missing values were not included in the dataset. Care was taken to ensure only the training set was used to calculate these mean values and for the standardisation process, to prevent data leakage.

The dataset is a matrix of numerical values, consisting of 11,801 features (columns) and 31,552 genes (rows). These values were used as inputs into model training. In the case of the random forest model, the model output would be a binary (0 indicating absence of the class, 1 indicating presence of the class) value for predicting categorical features, and a continuous value for predicting continuous features.

Since our dataset has many features, overfitting during model training can be a problem. One way to reduce this problem would be to perform feature selection and/or dimensionality reduction. However, this may not always be necessary as some models, such as random forest do demonstrate resistance to overfitting due to sample bootstrapping and node splitting.

### Machine learning, time trial

To determine a suitable machine learning model which gives a good balance of performance and time taken to train, we tested logistic regression, random forest, balanced random forest, linear support vector machine (SVM), and adaboost.

To improve the model performance and estimate the time it takes to analyse all features, we set out to identify suitable hyperparameters. The range of hyperparameters tested for the time trial is given in Table 1.

**Table 1:** HPs tested for time trial.

Model	Hyperparameter	Range of values
Adaboost	n_estimators (maximum number of estimators in model)	100, 120, 130, 150, and 200
	learning_rate (weight applied to each classifier at each training iteration, a higher learning rate increases the contribution of each estimator)	0.6, 0.625, 0.65, 0.675, and 0.7, 0.725, 0.75, 0.775, and 0.8
Balanced random forest	max_features (number of features to consider when looking for the best split in the tree)	sqrt, 0.1, 0.2, 0.3, 0.4, 0.5, and 0.75
	n_estimators (maximum number of estimators in model)	50, 100, 200, 500, and 1,000
	max_depth (maximum depth of the tree)	10, 20, 50, 70, 100, 125, 150, 200, 500, and None
Logistic regression	C (inverse of regularization strength, smaller values specify stronger regularization)	0.0001, 0.001, 0.01, 0.1, 1, 10, 100, 1,000, and 10,000
Linear SVM	C (inverse of regularization strength)	0.0001, 0.001, 0.01, 0.1, 1, and 10, 100, 1,000, and 10,000
Random forest	ccp_alpha (complexity parameter, used to determine extent of tree pruning)	0, 0.1, 0.001, and 0.001
	max_features (number of features to consider when looking for the best split in the tree)	sqrt, 0.1, 0.2, 0.3, 0.4, 0.5, and 0.75
	n_estimators (maximum number of estimators in model)	50, 100, 200, and 500
	max_depth (maximum depth of the tree)	20, 50, 100, 200, and None

We used a randomised search with 10 iterations within a nested 5-fold cross-validation approach. In a *k*-fold nested cross validation approach, the inner *k*-fold is used for hyperparameter optimization during random search, while the outer *k*-fold fold is used to test the model, hence the equivalent of k test/validation sets would be used. Such an approach was used to train the model with hyperparameter optimization, so as to minimise data leakage.^3^ The F1 metric was used to score models due to unbalanced sample sizes between the positive and negative classes. Genes labelled with the specific feature used as the class label are in the positive class, while genes which do not have that feature, are in the negative class. Before model training, for the specific GO term used as the class label, all parent and child GO terms related to that class label were removed. Parent and child GO terms are identified using GOATOOLS (Klopfenstein et al., 2018). Parent and child GO terms need to be removed to prevent data leakage during model training, as GO terms used as class labels are associated with their corresponding parent and child GO terms. This approach was also used for downstream machine learning applications, and modifications from this approach will be specified.

### Identification of optimal hyperparameters for random forest

To find the optimal hyperparameters for the random forest models, we chose 71 GO terms (Supplementary Table S5), that contained between 5 and 1,000 genes. The set of random forest hyperparameters tested is given in Table 1. The Out of Bag (OOB) F1 metric was used to score models. We used random search to estimate the optimal hyperparameters with 20 iterations for each GO term. We divided the OOB scores into three groups: high (OOB F1 ≥ 0.7), medium (0.5 ≤ OOB F1 < 0.7), and low (OOB F1 < 0.5) scoring.

To identify hyperparameters that were frequently found in the high performing group, we used two approaches. In the first one, we selected the most frequently occurring hyperparameter value. In the second, we selected the most frequently occurring group of values. Hyperparameters chosen from these two methods were used to train random forest models for the 71 GO classes. Additionally, default hyperparameters, and hyperparameters optimised for each GO class were also used. The selected hyperparameter values were tested on both original and shuffled data obtained by randomly shuffling the dataset columns.

### Construction of models for 9,535 features

To construct models for each feature, each feature would be selected as the prediction target and the remaining features would be used as the training features (the dataset). For GO terms, we removed child and parent features of a GO term that is used as a prediction target (label). GO terms with <10 genes were not used as targets. A total of 9,535 features were used as prediction targets in our workflow. The OOB F1 score was the metric used for categorical features, while the OOB *R*^2^ score was the metric used for continuous features. We used the hyperparameters that resulted in best overall performance, as estimated in “Identification of optimal hyperparameters for random forest”: ccp_alpha = 0.001, max_features = 0.2, n_estimators = 50, and max_depth = 200.

### Feature importance network construction

Construction of the network utilised the concept of calculating mutual ranks between feature pairs. To remove poorly predictable features, only features which scored ≥0.4 with the OOB F1 (for categorical features) or *R*^2^ (for continuous features) metric were selected for mutual rank calculations, which left 1,475 features. For these predicted features, all their nonzero feature importance values were converted into ranks, with the feature with the largest feature importance value given a rank of one, and the feature with the smallest feature importance value given the largest rank. The mutual rank of each pair was then calculated by taking the geometric mean of both ranks, as described in ATTED-II (Obayashi et al., 2009). The formula for calculating mutual ranks is given here:


MR(AB)=√(Rank(A→B)∗Rank(B→A))

In the formula, MR stands for mutual rank, MR(AB) refers to the MR of features A and B, and Rank(A → B) refers to the feature importance rank which A has with respect to B. Rank(B → A) would refer to the inverse.

To find a MR cut-off, the top 10% of the MR values (53,080 MR values were obtained corresponding to 53,080 feature pairs) were used to build the network, comprising 1,342 nodes and 5,308 edges. Edge weights were created by inverting a list of feature pairs, sorted by mutual ranks. An inversion is done since smaller mutual ranks indicate a stronger link between features, whereas a larger edge weight indicates the same idea. As such, the constructed network is a network of features, with putative biological links between them depicted by edges.

### Feature importance network analysis

The feature importance network was analysed to identify biologically relevant groups of features. Overall network metrics, which are betweenness centrality, clustering coefficient and degree, were calculated using Cytoscape 3.8.2 (Shannon et al., 2003).

A permutation test was used to identify statistically significantly associated feature categories, which are defined as each row of the table given in Table 2. First, the number of edges between all possible feature category pairs was calculated. Next, the features in all feature category pairs were shuffled 10,000 times, and we compared the number of edges after shuffling, with the number of edges before shuffling (the original number of edges). The calculated empirical value of *p* indicates whether the original number of edges is significantly depleted as compared to random chance. *p* values were corrected using the Benjamini–Hochberg correction (Benjamini and Hochberg, 1995).

**Table 2:** Summary of features used.

**Feature type**	**Feature name (number of features)**	**Feature purpose**
Gene expression	SPM (9)	Expression specificity
	TPM (6)	Gene expression levels
	DGE (436)	Differential gene expression
	Diurnal (13)	Diurnal gene expression, amplitude and time point
Gene family	Orthogroups (2)	Gene family size
Phylostrata	Phylostrata (1)	Phylostrata which genes belong to
Genomic information	Single copy genes (1)	Single copy genes in the same gene family
	Tandemly duplicated genes (1)	Tandemly duplicated genes in the same gene family
Protein domain	MobiDBLite (1)	Prediction of disordered domains regions
	Pfam (2761)	Collection of protein families
	TMHMM (1)	Prediction of transmembrane helices
	Number of domains (2)	Number of protein domains
Biochemical	Length of peptide (1)	Shows how long each peptide is
	Molecular weight (1)	Molecular weight of peptide
	Isoelectric point (pI) (1)	pI of peptide
PPI	Network centrality (2)	Degree and betweenness centrality
	Network clusters (1295)	Cluster size and ID
Gene coexpression	Network centrality (2)	Degree and betweenness centrality
	Network clusters (279)	Cluster size and ID
GO terms	GO terms (3645)	Experimentally determined gene annotations
cis-regulatory elements	cis-regulatory element names (82)	Gene regulation
	cis-regulatory element families (15)	Gene regulation
Multi-omics	GWAS (33)	Genomic loci within genes, correlated with phenotype traits
	TWAS (28)	Gene expression level, correlated with phenotype traits
Gene regulatory network	Network centrality (2)	Degree and betweenness centrality
	Network clusters (55)	Cluster size and id
	Properties (76)	Biological characteristics of transcription factors and their target genes
Aranet gene-interactions	Network centrality (2)	Degree and betweenness centrality
	Network clusters (2957)	Cluster size and id
Evolution	Homologs (22)	Presence of *A. thaliana* homolog in 22 species
	Nucleotide Diversity (1)	Nucleotide diversity calculated from *A. thaliana* accessions
Epigenetics	Gene body methylation (1)	Whether gene body is methylated
Conservation	Sequence conservation (3)	Protein sequence % identity to fungi, plants, and metazoans
	Percent identity to paralogs (1)	Maximum percent identity from BLAST to closest paralog
	dN/dS values (4)	dN/dS substitution rates between *A. thaliana* paralogs, and homologs from three plant species
	Paralog dS (1)	dS with putative paralog
PTMs	Protein PTM (58)	Protein PTM frequency

The first column describes the feature type. The second describes the feature name and parentheses indicate the number of features per name. The third column contains the feature description.

### Data analysis and availability

All data processing and analysis tasks, unless otherwise stated, used python 3.8.6 and its associated libraries for data science, such as pandas 1.1.4, numpy 1.19.4, scipy 1.6.1, and statsmodels 0.13.0. Networks were constructed and analysed using networkx 2.5 [Proceedings of the Python in Science Conference (2008): Exploring Network Structure, Dynamics, and Function using NetworkX], and analysis and visualisation of the feature importance network also made use of Cytoscape 3.8.2 (Shannon et al., 2003) and its associated apps. Machine learning was done by scikit-learn 0.23.2 (Pedregosa et al., 2011) while the balanced random forest model was trained using imbalanced-learn 0.7.0 (Lemaître et al., 2017). Data visualisation was done using matplotlib 3.4.2 (Hunter, 2007), seaborn 0.11.1 (Waskom, 2021), and ptitprince 0.2.5 (Allen et al., 2021).

The machine learning dataset, together with scripts used, are available from a github repository.^4^ Raw data used to create this dataset is available as Supplementary Data 1.

### Database development

The frontend is hosted on github and uses React.js and cytoscape.js (Franz et al., 2016). The frontend code is available as a github repository.^5^ The REST API backend uses python flask which retrieves data from Google Cloud Storage. The backend is hosted on Google App Engine.

## Results

### Assembly of 11,801 features for 31,552 Arabidopsis genes

We used 11,801 features for 31,522 *A. thaliana* genes, drawn from a wide variety of 37 categories (Supplementary Table S1; Table 2). Gene expression features include SPM, TPM, and differential gene expression (DGE) features. Gene SPM values (expression specificity, indicating whether a gene is, e.g., specifically expressed in roots) were obtained from nine organs, stem, female, male, leaf, flower, seeds, root, apical meristem, and root meristem. From TPM values from these organs, six summary statistics were calculated, which are mean, median, maximum, minimum, and variance calculated by square of SD and variance calculated by median absolute deviation divided by the median (MAD). DGE features were derived from 218 conditions, and each condition was used to create two features, which are the up and downregulated status for genes.

Genomic and evolutionary features include gene family (orthogroup), phylostrata, protein domains, gene regulation, and homolog features. One type of gene family size was calculated by counting the number of *A. thaliana* genes in the gene family, whereas the second type was calculated by counting the number of genes from all species in the gene family. For each orthogroup, its last common ancestor was assigned as its phylostrata. One method of counting the number of protein domains counts the total number of domains in each gene, while the other counts the total number of unique domains. Seventy six features describing biological characteristics of transcription factors (TFs) and their target genes (TGs) were also obtained from the gene regulatory network. An *A. thaliana* gene is defined to have a homolog with a particular species if that species has a gene in the same orthogroup as that *A. thaliana* gene.

To conclude, we ended up with 9,535 features as targets, as 2,266 GO term features were removed as they had <10 genes per term. As such, 11,801 features were used in our machine learning workflow to predict 9,535 targets.

### Finding the optimal machine learning model

To identify which machine learning method produces the most accurate predictions, we tested five methods [logistic regression, random forest, balanced random forest, linear support vector machines (SVM), and adaboost] and used the F1 score, which is the harmonic mean between precision and recall, to score the models. In addition, due to the resource intensiveness of the pipelines, we also noted the training time needed for finishing model training. For the time trial, 16 well annotated GO cellular component terms were used as labels (i.e., prediction targets; Supplementary Table S2). Both logistic regression and linear SVM produced warnings as their algorithms could not converge with the specified number of iterations (a hyperparameter), thus their results were not reliable.

Therefore, a second time trial was conducted, to determine the number of iterations needed for convergence and the time taken. Two GO classes, GO:0016020 and GO:0005829, were used together with all five models, with 10 random search iterations and 5-fold nested cross-validation. This would allow for a fair comparison of the time taken of all five models to be made, as a suitable number of iterations for logistic regression and linear SVM was used to ensure convergence. Using only two GO classes would help to ensure that all models were trained in a reasonable amount of time.

**Figure 1:**
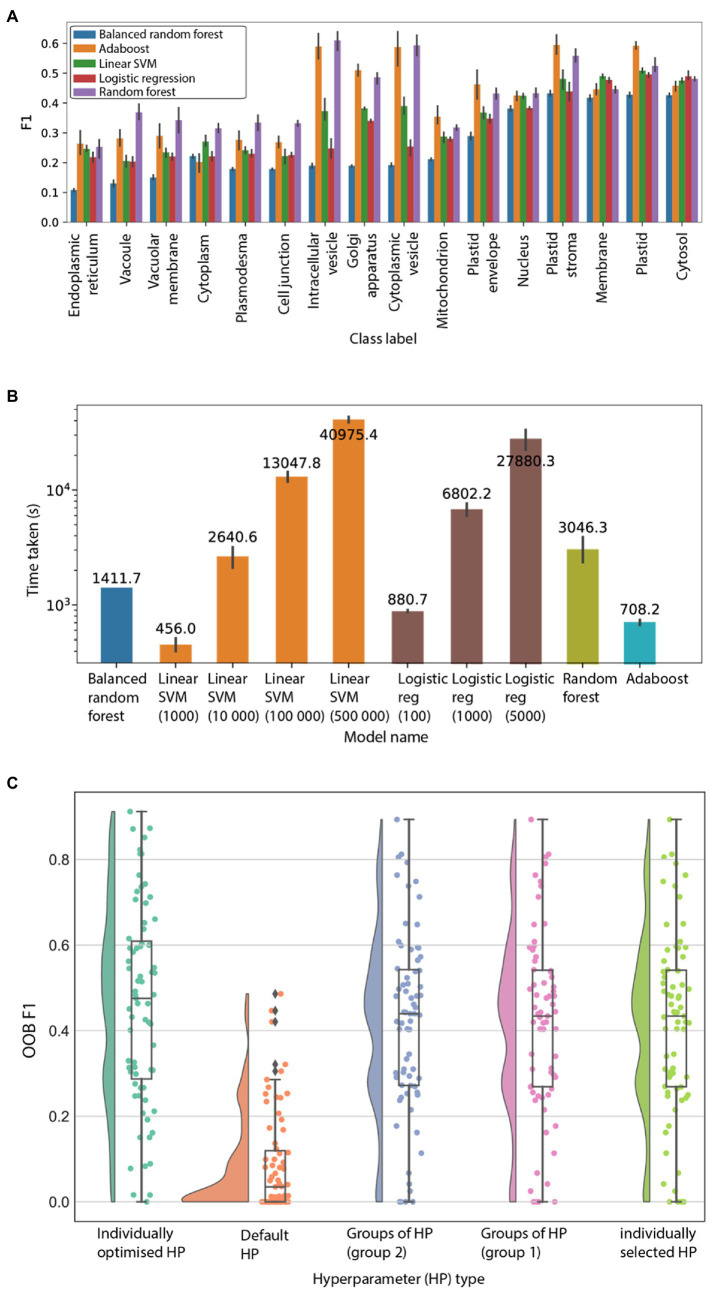
Evaluation of machine learning algorithms. **(A)** F1 scores (*y*-axis) of 16 GO cellular location terms (*x*-axis). The algorithms are logistic regression (average F1 score 0.32), balanced random forest (0.26), adaboost (0.41), random forest (0.43), and linear SVM (0.35). The predictions were performed five times, and the error bars represent the 95% CI. **(B)** Time (*y*-axis) taken to train the different machine learning models to finish training. The predictions were performed five times on GO terms GO:0005829 (cytosol) and GO:0016020 (membrane). **(C)** OOB F1 score (*y*-axis) for the 71 GO terms using different sets of hyperparameters. “Individually selected HP” refers to random grid search to optimise hyperparameters for each GO term individually. “Default HP” means that default hyperparameters are used. “Groups of HP (group 1)” and “(group 2),” refers to the most frequent hyperparameter group observed after optimizing HPs for the 71 GO terms. “Most frequent individual hyperparameter” refers to the most frequent individual hyperparameter chosen after optimizing for the 71 GO terms.

We observed that the random forest showed consistently high F1 scores (Figure 1A), and a reasonable amount of time to train (Figure 1B; Supplementary Figure S1; Supplementary Table S3). In addition, random forests allow one to use the out-of-bag (OOB) score to test the model, hence saving time by removing the need for a test/train split.

Machine learning models can be further tuned by adjusting their hyperparameters. To determine the suitable hyperparameters for the random forest model by considering model scores and training time, we tested the influence of four hyperparameters on the prediction performance of 71 GO labels (Supplementary Table S5). These are cost-complexity pruning (ccp_alpha), maximum number of features for each split in the tree (max_features), number of trees in the forest (n_estimators), and maximum depth of the tree (max_depth). We investigated four methods to identify the best hyperparameters. The first method identified which individual hyperparameter values are most frequently found among the best-performing models of the 71 GO terms. The second method identified the most frequently occurring groups of hyperparameters. The third method used the default hyperparameters (which are ccp_alpha = 0.0, max_features = auto, n_estimators = 100, and max_depth = none). The fourth method used hyperparameters individually optimised for each GO term, which represents the most computationally intensive approach to estimate the hyperparameters, as each model has to be optimised individually with cross-validation (Supplementary Tables S6, S7).

The outcome of the analysis revealed that the individually optimised hyperparameters produced the best-performing models (Figure 1C; average F1 = 0.46). Conversely, the default hyperparameters performed the worst (Figure 1C; average F1 = 0.09), showing that optimised hyperparameters can dramatically improve the performance of the models. Furthermore, two most frequent groups of parameters (group 1 and 2, average F1 scores of 0.41505 and 0.41536 respectively) and most frequent individual HP (F1 0.41504) performed comparably to the individually optimised HP (Figure 1C). These results show that “individually selected” and “groups” of hyperparameters perform very similarly to “individually optimised” hyperparameters. Based on these results, an “individually selected” hyperparameter values method was chosen to build models for each of the 9,535 features. To verify that our random forest workflow was able to perform better than random chance, we used the found HPs on both original and randomly shuffled data. Results show that our workflow with the selected hyperparameters performs better than random chance (Supplementary Figure S2) and hence were used for model training on all features.

### Calculating the predictive performance of biological features

**Figure 2:**
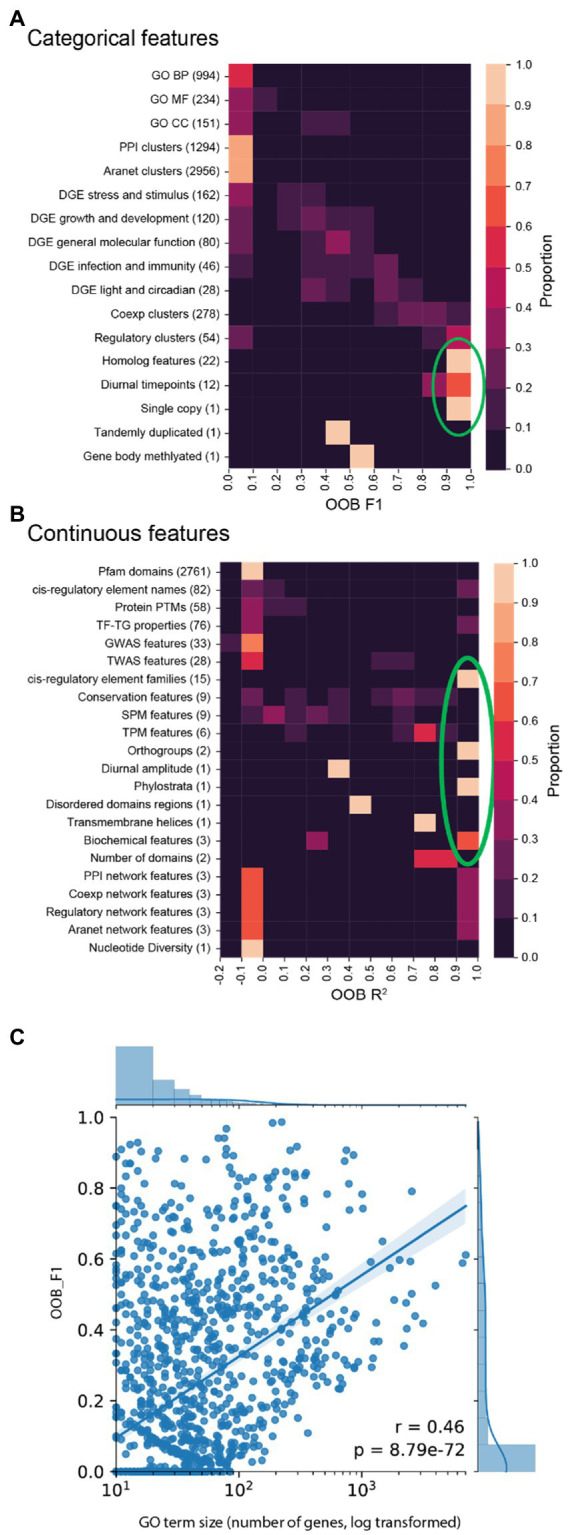
OOB F1 or *R*^2^ scores (*x*-axis) of the biological features. **(A)** F1 scores (*x*-axis) for categorical features (*y*-axis). **(B)** *R*^2^ continuous feature scores. **(C)** F1 scores (*y*-axis) of GO terms vs. number of genes in each GO term (*x*-axis).

To investigate which biological features can be predicted well by our machine learning model, for each of the 9,535 features we built a random forest model. To score the performance of each model, we used the OOB F1 score for categorical features (0 and 1 represent poor and perfect performance, respectively), while for continuous features we used the OOB R^2^ score (<0, 0, and 1 indicate predictions worse than always predicting the mean value of the target, poor performance (predict mean value of the target regardless of input) and perfect performance, respectively, Figure 2).

We set out to investigate how well the different types of features could be predicted. Features that could be predicted well (defined as lightly coloured squares to the right of the clustermaps and indicated by the green circles, in Figures 2A,B) comprise of homolog features, diurnal timepoints, single copy, cis-regulatory element families, orthogroups, phylostrata, and biochemical features. Conversely, features that could not be predicted well are all other features. While most GO terms and DGE are not accurately predicted, some of them have high scores (Figure 2A). To determine if the number of genes in each GO term influences model scores, a plot of scores against the number of genes was made (Figure 2C). This scatterplot showed a moderate but statistically significant relationship between the number of genes and OOB F1 score (Pearson’s *r* = 0.46, value of *p* = 8.79e−72). This indicates that increasing the number of genes in GO terms does positively influence model performance. Thus, the performance of machine learning models will be improved by the inclusion of more biological data for more genes.

### Construction of feature importance network

To investigate the biological relationships between features, we identified which features are mutually predictive of each other, and used this observation to infer biological relationships between them. To do this, we constructed a Feature Importance Network (FIN), where nodes represent machine learning features and edges represent features with putative biological relationships. To obtain the FIN, mutual ranks of feature pairs were calculated and the top 10% of them were used to construct the network. This is based on the assumption that these represent features that are mutually predictive of each other, which implies biological relationships between them. A total of 1,475 nodes (features) and 53,080 edges (mutual ranks) were present before applying the top 10% cutoff and after applying it, 1,342 nodes and 5,308 edges were selected to build the FIN.

The top 10% cut-off of mutual ranks was used similarly to other studies on gene expression (Mutwil et al., 2010). The threshold also resulted in a good balance between the number of edges in FIN, and the number of nodes with connections (Supplementary Figure S5). At 10% cut-off, most nodes (features) are connected, with the fewest edges, resulting in a compromise between the readability and richness of information.

**Figure 3:**
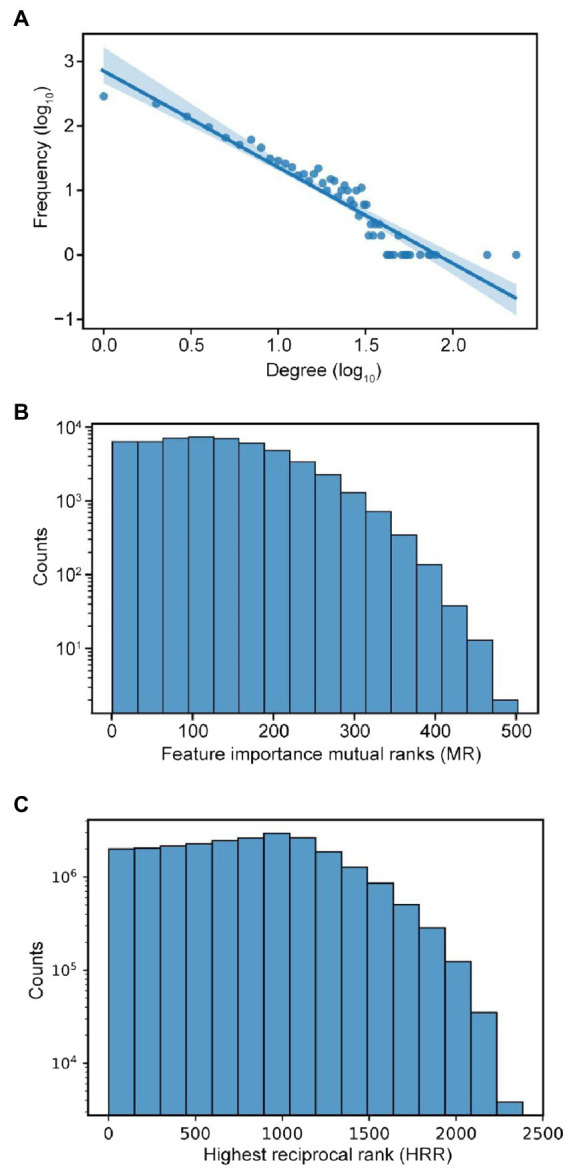
Characteristics of feature importance network. **(A)** Power-law distribution of node degrees. **(B)** Distribution of mutual ranks (MR) of feature importance values. **(C)** Distribution of highest reciprocal rank (HRR) of gene coexpression values in the coexpression network used in our study.

To analyse the topology of the network, we first investigate the node degree of the FIN, and we observed a power-law distribution (Figure 3A). This is consistent with a scale-free network topology observed in many biological networks such as metabolic (Kim et al., 2019), RNA (Panni et al., 2020), protein (Pastor-Satorras et al., 2003), and gene coexpression (van Noort et al., 2004) networks. The distribution of the mutual ranks (Figure 3B) is like the HRR distribution of the gene coexpression data used in our study (Figure 3C). Given that gene coexpression networks are known to be scale-free (Emamjomeh et al., 2017), this observed similarity can lend support to our inference of the scale-free nature of the FIN. Therefore, this network topology could imply that while many features are biologically linked to only a few others, a minority of features are biologically linked to many others.

**Figure 4:**
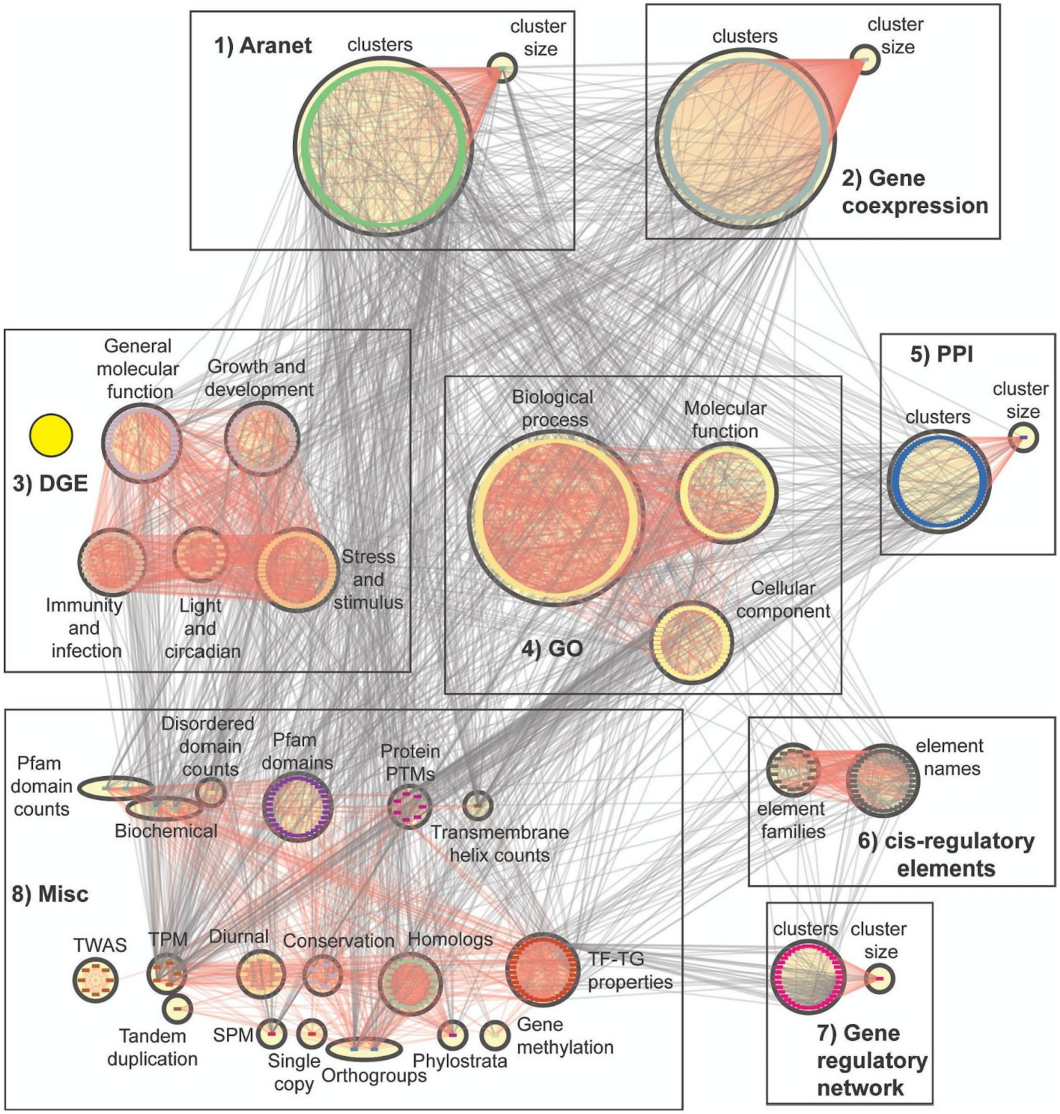
Feature importance network. Nodes represent features while edges connect features that have putative biological links. The features are divided into eight major groups (Table 2). Red edges show relationships within groups belonging to the same box. These groups are divided into eight boxes, and the first seven boxes represent clusters of feature categories. The boxes are 1 (Aranet features: cluster IDs and size), 2 (gene coexpression features: cluster IDs and size), 3 (DGE features: subdivided into five categories), 4 (GO terms: subdivided into three categories), 5 (PPI features: cluster IDs and size), 6 (cis-regulatory elements), 7 (gene regulatory features: cluster IDs and size), and 8 (a miscellaneous group which contains all other feature categories). Yellow circle highlights DGE features, showing how many of them are linked to each other, while also having links to other features.

To visualise the FIN, we grouped the nodes (features) according to their corresponding categories (Table 2). While we observed many edges between the features of the same category, we also observed many edges between features from different categories (Figure 4). An example of such an observation is in the DGE features (yellow circle, Figure 4), where they share many biological relationships with one another, while also having relationships with other feature types. The complex web of interactions between the features shows a complex relationship between the features of Arabidopsis genes.

**Figure 5:**
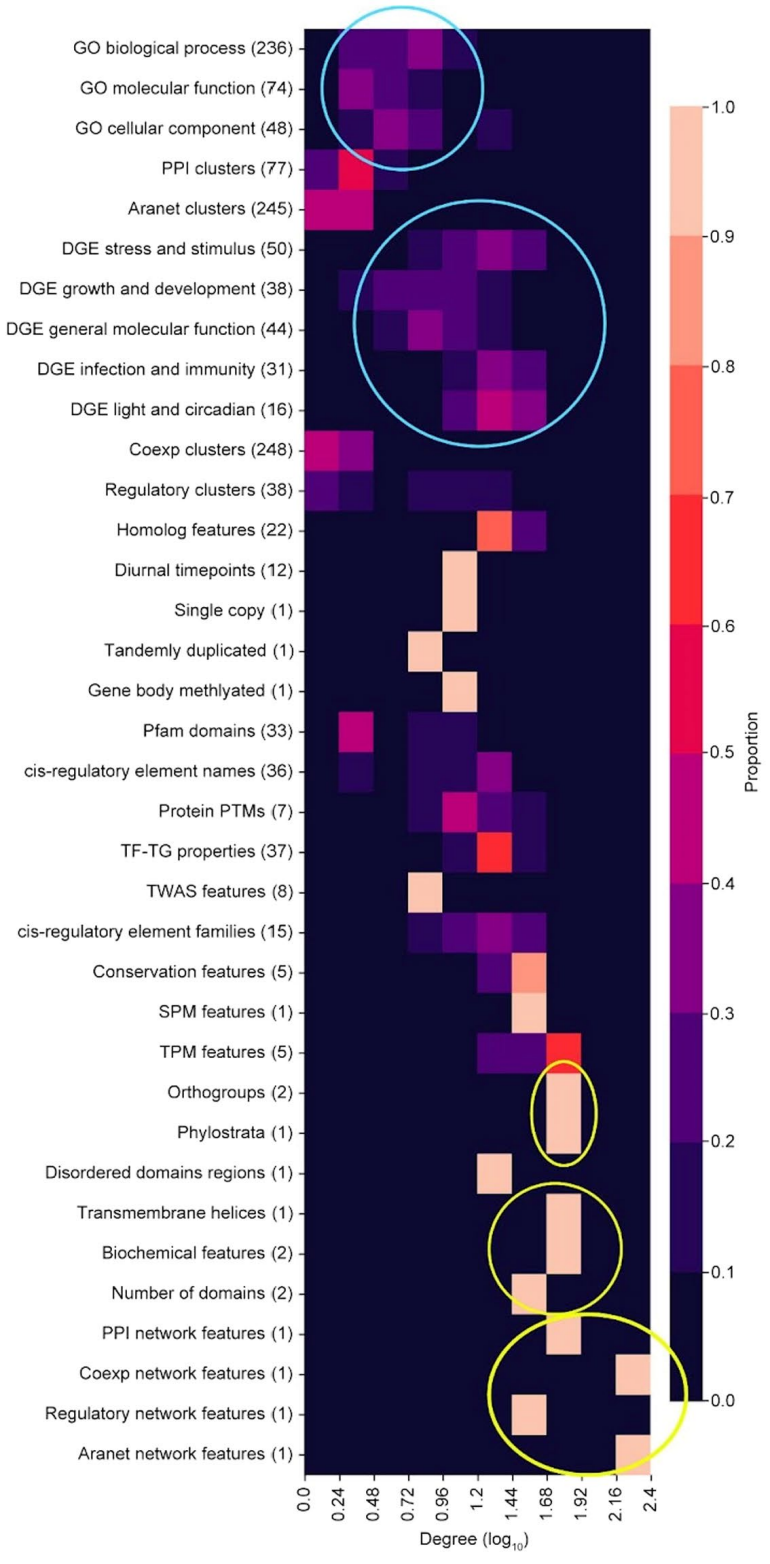
Degree distribution of feature categories. Top blue circle shows GO terms while the bottom blue circle shows DGE features. Top yellow circle shows orthogroups and phylostrata, middle yellow circle shows transmembrane helices, biochemical features (length and molecular weight of peptide) and number protein domains, and bottom yellow circle shows network features (cluster size) from the PPI, coexpression, regulatory and Aranet networks.

The node degree distribution of the feature categories in the FIN varies across categories (Figure 5). GO terms are observed to have low node degrees in general (top blue circle, Figure 5), while DGE features have a comparatively higher node degree distribution (bottom blue circle, Figure 5). This implies that GO terms tend to be less functionally linked to other features, while DGE features exhibit more functional links. Orthogroup and phylostrata (top yellow circle), transmembrane helices, biochemical features (length and molecular weight of peptide) and number protein domains (middle yellow circle), and network features (bottom yellow circle), are feature categories which exhibit high node degree distributions (Figure 5). This implies that they share functional links with many other features. The network features with a high node degree distribution are the cluster size feature of their respective biological networks, which are connected to many cluster ID features (Box 1, 2, 5, and 7 in Figure 4).

### Identification of functionally-related feature categories

**Figure 6:**
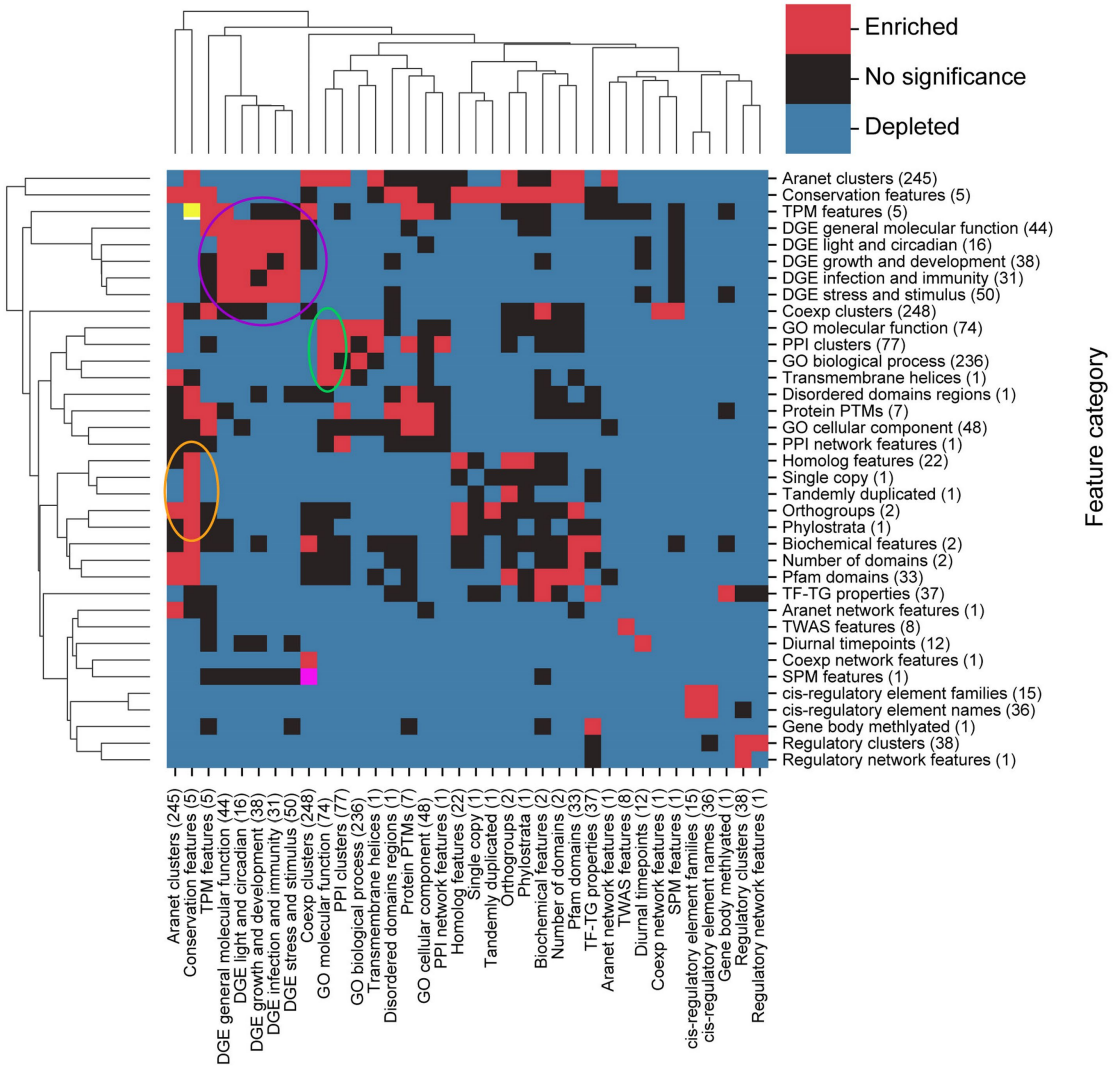
Identification of significantly associated feature types. The clustermap shows whether there are significantly (BH-adjusted values of *p* < 0.05) more (red squares) or significantly less (blue square) between feature categories that are expected by chance. Not statistically significant associations are indicated by black boxes. Circles indicate clusters of feature categories which are associated with each other. Yellow (top left) and pink (bottom left) squares are red squares which have been coloured differently to enable easy identification when our study refers to them.

To identify which feature categories are significantly linked to each other, we determined which categories have more edges between them than expected by chance. This was achieved by performing a permutation test on the number of edges linking categories from the FIN together and comparing the permuted number with the original number of edges (Figure 6).

Most pairs of feature categories have a significantly smaller number of connections between them than expected by chance (Figure 6, blue squares), indicating that they are less likely to be functionally related. The exceptions are five DGE groups (purple circle), GO molecular function, PPI clusters, GO biological process and transmembrane helices (green circle), conservation features (conservation of gene sequences), homologs, single copy, tandemly duplicated, orthogroups, and phylostrata (orange circle), that represents clusters of significantly connected feature categories (Figure 6). Conservation and TPM features (yellow square, top left in Figure 6) and coexpression clusters and SPM features (pink square, bottom left in Figure 6) are pairs of features which are also seen to be linked.

The number of biological relationships within feature categories also tends to be higher than expected, as depicted by the many red squares along the clustermap diagonal. Some black squares are also observed along the diagonal, which indicates that the number of relationships within their corresponding feature categories is not statistically significant. Taken together, features tend to share functional links within the same category, compared to across categories.

### Construction of finder.plant.tools—online database to browse FIN

To provide a user-friendly interface for scientists to explore the FIN and identify biologically associated features, we created an online database,^6^ finder.plant.tools that can be queried by feature names. The database can display the local FIN neighbourhoods of features. To represent positively or negatively correlated features, and the strength of the correlations, we use red edges to indicate positive association, and blue edges indicate negative association. Grey edges indicate associations between neighbours which do not involve the target feature. The edge width indicates the strength of association (based on mutual rank), where a thicker width indicates a stronger association.

**Figure 7:**
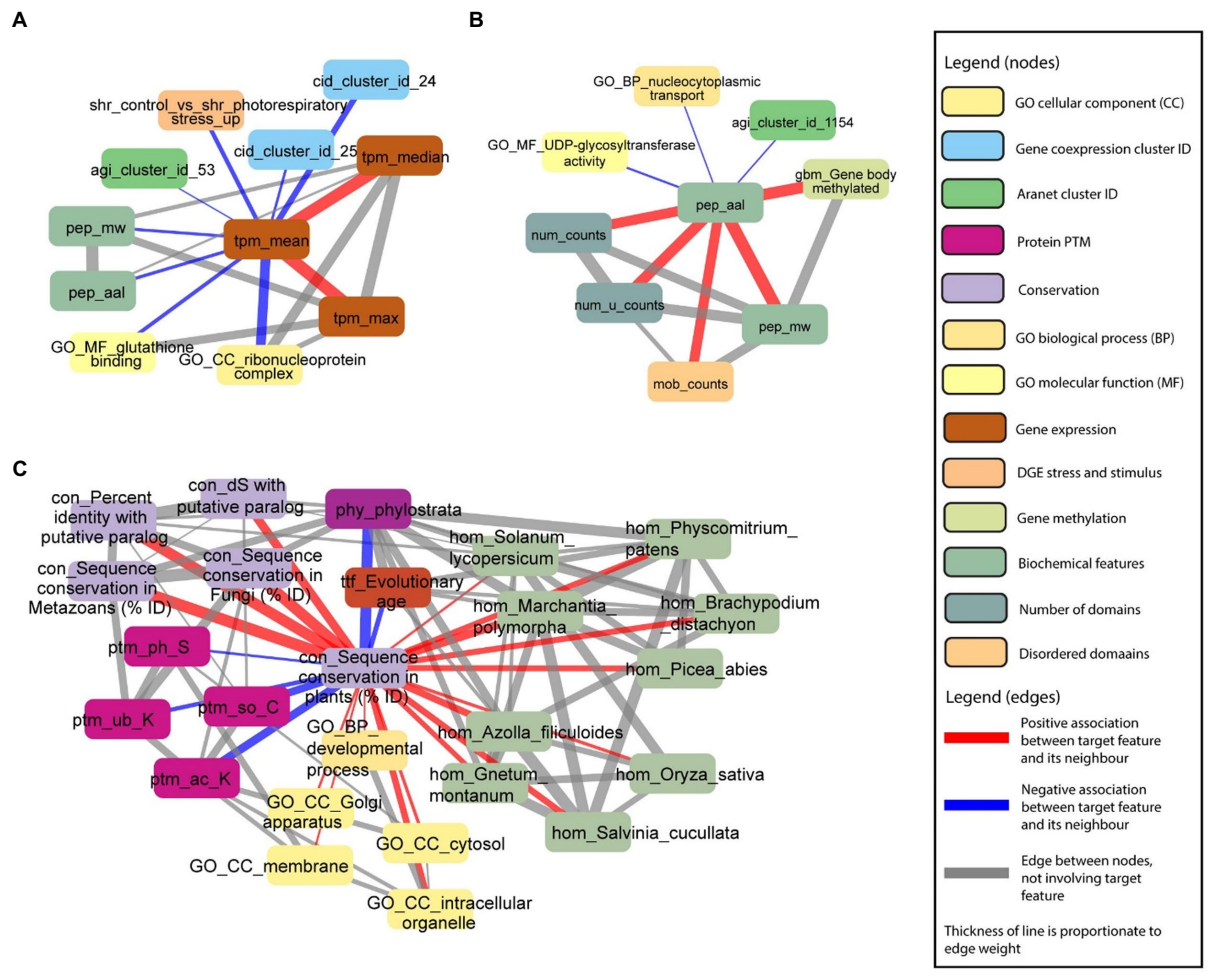
Examples capturing protein sizes and conservation. Selected nodes and edges from the local neighbourhood of specific features in the database are shown. **(A)** Mean gene expression (tpm_mean) is positively associated with maximum (tpm_max) and median (tpm_median) gene expression. **(B)** Protein length (pep_aal) is positively associated with the number of protein domains (num_counts), number of unique protein domains (num_u_counts), number of disordered protein domains (mob_counts) and protein molecular weight (pep_mw). **(C)** Sequence conservation in plants [con_Sequence_conservation_in_plants_(%_ID)] is positively associated with sequence conservation in paralogs, fungi and metazoans (nodes starting with “con_”), fundamental GO terms (nodes starting with “GO_”), and homology with multiple plant species (nodes starting with “hom”). Sequence conservation in plants is negatively associated with the evolutionary age of target genes of transcription factors (ttf_Evolutionary_age), phylostrata (phy_phylostrata), and protein PTMs (nodes starting with “ptm_”).

To demonstrate the ability of the FIN database to capture biologically relevant information, we set out to investigate several features with expected associations. First, we observed that mean gene expression (tpm_mean, brown rounded rectangle) is positively associated with maximum (tpm_max, brown rounded rectangle) and median (tpm_median, brown rounded rectangle) gene expression (Figure 7A). This indicates that genes with higher mean expression tend to have higher median and maximum expression. Conversely, we observed a negative association between mean gene expression, and protein molecular weight (pep_mw, green rounded rectangle) and length (pep_aal, green rounded rectangle). This indicates genes with lower mean expression, tend to code for proteins with a lower molecular weight and have a shorter length. Furthermore, we observed that genes belonging to co-expression clusters 24 and 254 (cid_cluster_id_24 and 254, blue rounded rectangles), genes belonging to the ribonuceloprotein complex (GO_CC_ribonucleoprotein complex, beige rounded rectangle), and genes involved in glutathione binding (GO_MF_glutathione binding, yellow rounded rectangle), tend to have lower mean expression.

In the second example, we observed that protein length is positively associated with the number of protein domains (num_counts, greenish beige rounded rectangle), number of unique protein domains (num_u_counts, greenish beige rounded rectangle), number of disordered domains (mob_counts, beige rounded rectangle), and protein molecular weight (Figure 7B). Not surprisingly, proteins with a longer length tend to have a higher number of protein domains, more disordered domains, and a higher molecular weight. However, we also observed a link between methylation of the gene body and protein length, suggesting that longer proteins tend to be more regulated on the epigenetic level.

Finally, in the third example, we saw that sequence conservation in plants [con_sequence conservation in plants (% ID), light purple rounded rectangle] is positively associated with sequence conservation in paralogs (con_dS with putative paralog and con_percent identity with putative paralog, light purple rounded rectangle), fungi (con_sequence conservation in fungi (% ID), light purple rounded rectangle) and metazoans (con_sequence conservation in metazoans (% ID), light purple rounded rectangle), and homology with multiple plant species (green rounded rectangles on the right; Figure 7C). Conversely, sequence conservation in plants is negatively associated with the evolutionary age of genes captured by phylostrata (phy_phylostrata, dark purple rounded rectangles; Figure 7C). Phylostrata feature ranges from 1 (gene families found in Archaeplastida) to 21 (gene families only found in *A. thaliana*), and the negative association between sequence conservation and phylostrata can be explained by the fact that genes with higher conservation are older, and thus have a lower phylostrata number. Interestingly, sequence conservation in plants is negatively associated with protein post-translational modifications (PTM, ptm_ph_S: serine phosphorylation, ptm_so_C: cysteine S-sulfenylation, ptm_ub_K: lysine ubiquitination, and ptm_ac_K: lysine acetylation, purple rounded rectangles), indicating that younger proteins tend to be most post-translationally modified. Taken together, these examples reveal a mixture of expected and novel insights, indicating that the FIN can be used to gain new knowledge about the molecular wiring of Arabidopsis.

**Figure 8:**
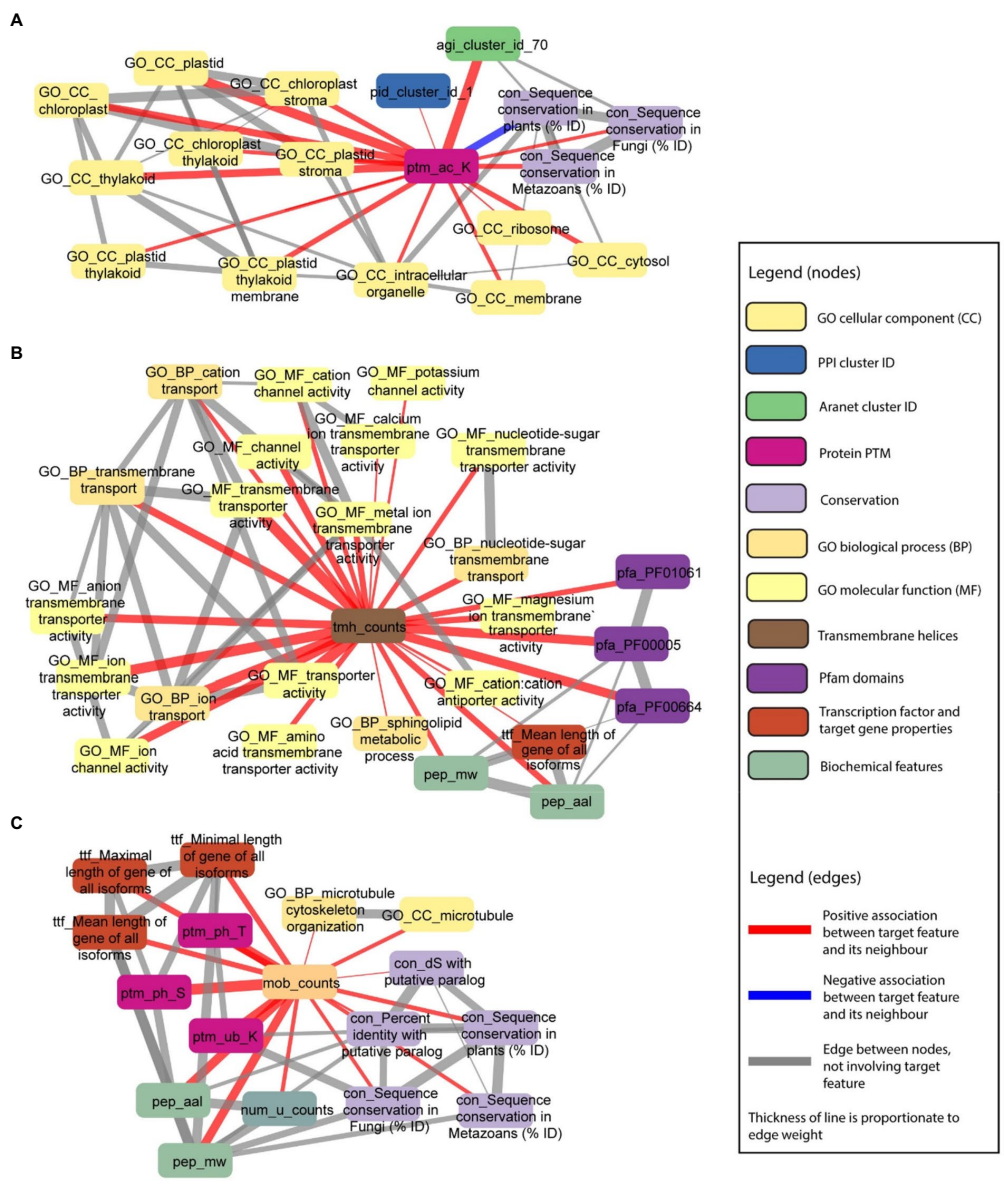
Examples capturing posttranslational modifications and the number of transmembrane and disordered domains. Selected nodes and edges from the local neighbourhood of specific features in the database are shown. **(A)** Protein PTM—lysine acetylation (ptm_ac_K) is positively associated with GO cellular components terms (nodes starting with “GO_CC_”), especially those related to the chloroplast. **(B)** Number of transmembrane helices in a protein sequence (tmh_counts) is positively associated with GO transmembrane transporter and channel terms (nodes starting with “GO_”), and sphingolipid metabolic process (GO_BP_sphingolipid metabolic process). **(C)** Number of disordered domains in a protein sequence (mob_counts) is positively associated with sequence conservation in paralogs, plants, fungi and metazoans (nodes starting with “con_”), GO microtubule terms (nodes starting with “GO_”), gene length (nodes starting with “ttf_”), protein PTMs (nodes starting with “ptm_”), protein length (pep_aal), number of unique protein domains (num_u_counts), and protein molecular weight (pep_mw).

We set out to investigate more complex examples. First, we observed that post-translational modification of lysine acetylation is positively associated with multiple GO cellular locations (e.g., GO_CC_thylakoid, GO_CC_chloroplast stroma, and GO_CC_chloroplast, beige rounded rectangles), especially those related to the chloroplast (Figure 8A), implying that lysine acetylation takes place in chloroplast, or is important for importing proteins into the chloroplast. Furthermore, we observed associations to Aranet cluster 70 (agi_cluster_id_70, green rounded rectangle) and PPI cluster 1 (pid_cluster_id_1, blue rounded rectangle), suggesting that proteins with this post-translational modification tend to physically interact.

In the second example, we observed that the number of transmembrane helices in a protein sequence (tmh_counts, brown rounded rectangle) is positively associated with multiple GO terms associated with channels (e.g., GO_BP_transmembrane transport, GO_MF_cation channel activity, and GO_MF_potassium channel activity, beige/yellow rounded rectangles), not surprisingly indicating that channels tend to have more transmembrane helices (Figure 8A). In addition, we also observed association to three Pfam domains (pfa_PF01061: ABC-2 type transporter, pfa_PF00005: ABC transporter, and pfa_PF00664: ABC transporter transmembrane region, purple rounded rectangles), which is in line with proteins with transporter domains having more transmembrane helices. Furthermore, there is a strong correlation between the number of transmembrane helices, molecular weight, and protein length, which can be explained by proteins having more domains (transmembrane helices, disordered domains, and others), being longer. Interestingly, we also observed an association to the sphingolipid metabolic process (GO_BP_sphingolipid metabolic process, beige rounded rectangles), suggesting that this metabolic process involves proteins with transmembrane helices (Figure 8B).

In the third example, we observed that the number of disordered domains in a protein sequence is positively associated with sequence conservation in paralogs, plants, fungi, and metazoans, suggesting that proteins with many disordered domains are of an ancient origin (Figure 8C). In line with the above examples in Figure 8B, the number of disordered domains is correlated with protein size (pep_aal: protein length, pep_mw: protein molecular weight, num_u_counts: number of unique protein domains). Interestingly, the number of disordered domains is positively correlated to several posttranslational modifications (ptm_ph_T: threonine phosphorylation, ptm_ph_S: serine phosphorylation, and ptm_ub_K: lysine ubiquitination, purple rounded rectangles), and the number of unique protein domains a protein has (num_u_counts). The latter suggests that multi-domain proteins tend to contain disorganised domains. Finally, we observed that of all biological processes captured by GO, microtubules tend to be the only one positively associated with the number of disordered domains.

## Discussion

Understanding biological relationships between molecular characteristics is critical to understanding how life works, and machine learning has great potential to contribute to achieving such an objective. Here, we analysed 31,522 *A. thaliana* genes using 11,801 features. These features are drawn from a wide variety of categories such as genomic, transcriptomic, evolutionary, biochemical, and protein and gene interactions. We applied a machine learning workflow using random forests and constructed a FIN that shows features with putative biological relationships.

We observed that it is important to optimise hyperparameters for increased machine learning performance as the models gained on average a 4.6-fold increase in performance after optimisation. Interestingly, rather than optimising parameters for each of the 9,535 models individually, which is computationally costly, hyperparameters optimised for a subset of 71 models resulted in similar performance. Since individual optimization takes a significantly longer time for training compared to using fixed hyperparameters, we chose the most frequently occurring, individually selected hyperparameter values for model training.

While the performance of most GO term and DGE feature models were not high, a minority had high scores (Figure 2A). We observed a significant positive correlation between the number of genes in each GO term and OOB F1 score (Figure 2C). This implies that poor machine learning performance could be due to the small number of genes in many of the GO features. A study by Rifaioglu et al. also observed a strong correlation between GO term size and performance. While studies predicting GO terms have reported higher scores (Kulmanov et al., 2018; Sureyya Rifaioglu et al., 2019; Littmann et al., 2021), these studies focused on GO terms with a larger number of genes through inclusion of computationally annotated GO terms as prediction targets (Littmann et al., 2021).

There is a lack of experimental GO terms for many Arabidopsis genes due to the labour intensive nature of experimental work. Conversely, each DGE experiment can identify hundreds or thousands of differentially expressed genes. Since machine learning tends to perform better with larger amounts of training data, this could explain why our DGE terms perform slightly better than GO terms in terms of machine learning prediction.

We observed that the FIN shows a power-law distribution (Figure 3), indicating that it is a scale-free network, which is typical for many biological networks, such as protein, metabolic, and coexpression networks (Clote, 2020). We observed that many features are connected to each other, highlighting the complex web of biological interactions involved in the molecular wiring of Arabidopsis (Figure 4). DGE features have a comparatively higher number of functional links to other features than GO terms (Figure 5), and many of them tend to be fellow DGE features (yellow circle, Figure 4). This suggests that different stimuli can activate similar differential gene expression programs, which is the basis for online tools such as AtCAST (http://atpbsmd.yokohama-cu.ac.jp/cgi/atcast/home.cgi; Sasaki et al., 2011). For example, the diverse stress factors that plants face often activate similar cell signalling pathways and cellular responses, such as the production of stress proteins and upregulation of the antioxidant machinery (Pérez-Clemente et al., 2013). Genes belonging to different orthogroups and phylostrata have been shown to be associated with organ-specific gene expression, gene functions such as cell cycle organisation and phytohormone action, and diverse abiotic stress responses (Mustafin et al., 2019; Julca et al., 2021).

While DGE features have a higher number of functional links to GO terms (Figure 5), most of the DGE features tend to be poorly predicted. This indicates that the high quantity of features that can explain DGE features does not result in a high quality of the predictions.

To provide access to the FIN, we constructed an online database, FINder, available at http://finder.plant.tools/. The examples generated by FINder showed expected biological relationships between features. For example, mean gene expression is positively associated with maximum and median gene expression (Figure 7A), which is expected as mean and median are measures of central tendency, hence both are expected to be positively associated. Furthermore, protein length is positively associated with the number of protein molecular weight, protein domains, and disordered regions (Figure 7B), which is expected as longer proteins can contain a greater number of domains and disordered regions. Furthermore, sequence conservation in plants is positively associated with sequence conservation in paralogs, fungi and metazoans, fundamental GO terms, and homology with multiple plant species (Figure 7C). This is an expected finding as the greater the degree of sequence conservation in plants, the more likely that the gene sequence is conserved throughout evolution. Furthermore, highly conserved genes are more likely to play key essential and fundamental functions, such as in developmental processes (Chen et al., 2012; Mustafin et al., 2019).

Interestingly, sequence conservation in plants is negatively associated with serine phosphorylation, cysteine S-sulfenylation, lysine ubiquitination, and lysine acetylation (Figure 7C). This indicates that younger proteins tend to be more posttranslationally modified than older proteins, which is supported by studies suggesting that the range of PTMs has increased throughout evolution (Beltrao et al., 2013; Narasumani and Harrison, 2018).

Further analysis of posttranslational modifications showed that lysine acetylation is positively associated with multiple GO cellular locations, especially those related to the chloroplast (Figure 8A). Lysines are found in the subcellular localization signal domains of proteins, and their acetylation can regulate protein subcellular localization (Kim et al., 2006; Choudhary et al., 2014). Lysine acetylation may be an important posttranslational modification in the chloroplast, as four Calvin cycle enzymes are acetylated (Finkemeier et al., 2011). Studies in strawberry (Fang et al., 2015), soybean (Li et al., 2021), rice (Xiong et al., 2016), tea leaves (Jiang et al., 2018), and wheat (Zhang et al., 2016), indicate that a large proportion of lysine-acetylated proteins are predicted to be localised to the chloroplast. Therefore, lysine acetylation associations identified by finder.plant.tools support these studies, and suggest that it plays a key role in chloroplast function.

We also observed associations between PTMs (threonine and serine phosphorylation, lysine ubiquitination) with the number of disordered domains (Figure 8C), which is in line with disordered regions in proteins being posttranslationally modified (Gao and Xu, 2011; Kurotani et al., 2014). The proportion of PTM sites was recently shown to be higher in the intrinsically disordered protein domains than the structured domains (Gao et al., 2021), where phosphorylation of serine and threonine, acetylation, and methylation were over-represented in disordered regions of seven species (animals, plants, and fungi). Interestingly, lysine ubiquitination is another PTM which we observed (Figure 8C), that to the best of our knowledge has not been documented in the literature as being tied to disordered regions.

Future work would involve extending this database to include FINs from other species. This would require the creation of multiple types of experimental data for them and would allow for cross-species comparison of FINs. Such a comparison would allow for a greater understanding of the similarities and differences in the molecular mechanisms underlying gene function across different plant species.

Due to the increasing amounts of biological data which is generated, future work could also involve expanding our machine learning dataset with new feature types. Given that machine learning techniques typically improve in accuracy when they are trained on more data, including a wider array of features from experimental sources, could identify further novel relationships between features.

Our findings can be used as a base for future studies that aim to predict relationships between specific sets of features. Our dataset comprises (to our knowledge) the most comprehensive collection of Arabidopsis gene features, which we envision will be invaluable in predicting the various aspects of gene function, and the relationships between genetic features.

### Conclusion

To conclude, we created a dataset of 11,801 features with 31,552 *A. thaliana* genes and used machine learning to propose functional links between the features. Feature importance values from our approach were used to create a Feature Importance Network (FIN), which revealed a variety of potentially significant biological relationships between different types of features. An online database, finder.plant.tools,^7^ was created to provide a user-friendly way of accessing the FIN.

## Data availability statement

The datasets presented in this study can be found in online repositories. The names of the repository/repositories and accession number(s) can be found in the article/Supplementary material.

## Author contributions

MM designed research. JN performed research. JN and SC constructed the finder database. All authors contributed to the article and approved the submitted version.

## Funding

MM is supported by a NTU Start-Up Grant and Singaporean Ministry of Education grant MOE2018-T2-2-053, while JN is supported by an NTU PhD stipend.

## Conflict of interest

The authors declare that the research was conducted in the absence of any commercial or financial relationships that could be construed as a potential conflict of interest.

## Publisher’s note

All claims expressed in this article are solely those of the authors and do not necessarily represent those of their affiliated organizations, or those of the publisher, the editors and the reviewers. Any product that may be evaluated in this article, or claim that may be made by its manufacturer, is not guaranteed or endorsed by the publisher.

## Footnotes

^2^ http://finder.plant.tools/

^3^ https://scikit-learn.org/stable/auto_examples/model_selection/plot_nested_cross_validation_iris.html

^4^ https://github.com/jonng1000/ml_plant

^5^ https://github.com/Sweekwang/golabel

^6^ http://finder.plant.tools/

^7^ http://finder.plant.tools/

## Supplementary material

SUPPLEMENTARY DATA 1 Machine learning dataset, zip file.

SUPPLEMENTARY TABLE S1 Complete feature table.

SUPPLEMENTARY TABLE S2 16 GO terms, along with their description, used for testing the scores of 5 ML models.

SUPPLEMENTARY TABLE S3 Summarising results of the first time trial experiment.

SUPPLEMENTARY TABLE S4 Table showing the number of classes chosen for hyperparameter optimisation, note that a few of them have < 5 chosen when there is < 5 which did not meet the criteria.

SUPPLEMENTARY TABLE S5 Table showing 71 GO terms, along with their description, used for hyperparameter optimization of random forest, to determine which is the best hyperparameter set for ML workflow development.

SUPPLEMENTARY TABLE S6 Frequency of individual HPs during HP optimisation across different score categories, excel sheet.

SUPPLEMENTARY TABLE S7 Frequency of groups HPs during HP optimisation across different score categories, excel sheet.

## References

[ref1] Allen M. Poggiali D. Whitaker K. Marshall T. R. Van Langen J. Kievit R. A. (2021). Raincloud plots: a multi-platform tool for robust data visualization. Wellcome Open Res. 4:63. doi: 10.12688/wellcomeopenres.15191.2, PMID: 31069261 PMC6480976

[ref2] Aromolaran O. Aromolaran D. Isewon I. Oyelade J. (2021). Machine learning approach to gene essentiality prediction: a review. Brief. Bioinform. 22:bbab128. doi: 10.1093/bib/bbab128, PMID: 33842944

[ref3] Athar A. Füllgrabe A. George N. Iqbal H. Huerta L. Ali A.. (2019). ArrayExpress update—from bulk to single-cell expression data. Nucleic Acids Res. 47, D711–D715. doi: 10.1093/nar/gky964, PMID: 30357387 PMC6323929

[ref4] Beltrao P. Bork P. Krogan N. J. van Noort V. (2013). Evolution and functional cross-talk of protein post-translational modifications. Mol. Syst. Biol. 9:714. doi: 10.1002/msb.201304521, PMID: 24366814 PMC4019982

[ref5] Benjamini Y. Hochberg Y. (1995). Controlling the false discovery rate: a practical and powerful approach to multiple testing. J. R. Stat. Soc. Ser. B Methodol. 57, 289–300. doi: 10.1111/j.2517-6161.1995.tb02031.x

[ref6] Berardini T. Z. Reiser L. Li D. Mezheritsky Y. Muller R. Strait E.. (2015). The Arabidopsis information resource: making and mining the “gold standard” annotated reference plant genome. Gene 53, 474–485. doi: 10.1002/dvg.22877, PMID: 26201819 PMC4545719

[ref7] Bray N. L. Pimentel H. Melsted P. Pachter L. (2016). Near-optimal probabilistic RNA-seq quantification. Nat. Biotechnol. 34, 525–527. doi: 10.1038/nbt.3519, PMID: 27043002

[ref8] Chang S. Lee U. Hong M. J. Jo Y. D. Kim J.-B. (2021). Time-series growth prediction model based on U-net and machine learning in Arabidopsis. Front. Plant Sci. 12:721512. doi: 10.3389/fpls.2021.721512, PMID: 34858446 PMC8631871

[ref9] Chen W.-H. Trachana K. Lercher M. J. Bork P. (2012). Younger genes are less likely to be essential than older genes, and duplicates are less likely to be essential than singletons of the same age. Mol. Biol. Evol. 29, 1703–1706. doi: 10.1093/molbev/mss014, PMID: 22319151 PMC3375470

[ref10] Cheng C.-Y. Li Y. Varala K. Bubert J. Huang J. Kim G. J.. (2021). Evolutionarily informed machine learning enhances the power of predictive gene-to-phenotype relationships. Nat. Commun. 12:5627. doi: 10.1038/s41467-021-25893-w, PMID: 34561450 PMC8463701

[ref11] Choudhary C. Weinert B. T. Nishida Y. Verdin E. Mann M. (2014). The growing landscape of lysine acetylation links metabolism and cell signalling. Nat. Rev. Mol. Cell Biol. 15, 536–550. doi: 10.1038/nrm3841, PMID: 25053359

[ref12] Clote P. (2020). Are RNA networks scale-free? J. Math. Biol. 80, 1291–1321. doi: 10.1007/s00285-019-01463-z, PMID: 31950258 PMC7052049

[ref13] Emamjomeh A. Saboori Robat E. Zahiri J. Solouki M. Khosravi P. (2017). Gene co-expression network reconstruction: a review on computational methods for inferring functional information from plant-based expression data. Plant Biotechnol. Rep. 11, 71–86. doi: 10.1007/s11816-017-0433-z

[ref14] Enright A. J. Van Dongen S. Ouzounis C. A. (2002). An efficient algorithm for large-scale detection of protein families. Nucleic Acids Res. 30, 1575–1584. doi: 10.1093/nar/30.7.1575, PMID: 11917018 PMC101833

[ref15] Fang X. Chen W. Zhao Y. Ruan S. Zhang H. Yan C.. (2015). Global analysis of lysine acetylation in strawberry leaves. Front. Plant Sci. 6:739. doi: 10.3389/fpls.2015.00739, PMID: 26442052 PMC4569977

[ref16] Finkemeier I. Laxa M. Miguet L. Howden A. J. M. Sweetlove L. J. (2011). Proteins of diverse function and subcellular location are lysine acetylated in Arabidopsis. Plant Physiol. 155, 1779–1790. doi: 10.1104/pp.110.171595, PMID: 21311031 PMC3091095

[ref17] Franz M. Lopes C. T. Huck G. Dong Y. Sumer O. Bader G. D. (2016). Cytoscape.Js: a graph theory library for visualisation and analysis. Bioinformatics 32, 309–311. doi: 10.1093/bioinformatics/btv557, PMID: 26415722 PMC4708103

[ref18] Fu Y. Xu J. Tang Z. Wang L. Yin D. Fan Y.. (2020). A gene prioritization method based on a swine multi-omics knowledgebase and a deep learning model. Commun. Biol. 3:502. doi: 10.1038/s42003-020-01233-4, PMID: 32913254 PMC7483748

[ref19] Gao C. Ma C. Wang H. Zhong H. Zang J. Zhong R.. (2021). Intrinsic disorder in protein domains contributes to both organism complexity and clade-specific functions. Sci. Rep. 11:2985. doi: 10.1038/s41598-021-82656-9, PMID: 33542394 PMC7862400

[ref20] Gao J. Xu D. (2011). Correlation between posttranslational modification and intrinsic disorder in protein. Biocomputing 2012, 94–103. doi: 10.1142/9789814366496_0010 PMC5120255 22174266

[ref21] Geng H. Wang M. Gong J. Xu Y. Ma S. (2021). An Arabidopsis expression predictor enables inference of transcriptional regulators for gene modules. Plant J. Cell Mol. Biol. 107, 597–612. doi: 10.1111/tpj.15315, PMID: 33974299

[ref22] Goodstein D. M. Shu S. Howson R. Neupane R. Hayes R. D. Fazo J.. (2012). Phytozome: a comparative platform for green plant genomics. Nucleic Acids Res. 40, D1178–D1186. doi: 10.1093/nar/gkr944, PMID: 22110026 PMC3245001

[ref23] Greener J. G. Kandathil S. M. Moffat L. Jones D. T. (2021). A guide to machine learning for biologists. Nat. Rev. Mol. Cell Biol. 23, 40–55. doi: 10.1038/s41580-021-00407-0, PMID: 34518686

[ref24] Hassani-Pak K. Singh A. Brandizi M. Hearnshaw J. Parsons J. D. Amberkar S.. (2021). KnetMiner: a comprehensive approach for supporting evidence-based gene discovery and complex trait analysis across species. Plant Biotechnol. J. 19, 1670–1678. doi: 10.1111/pbi.13583, PMID: 33750020 PMC8384599

[ref25] Hooper C. M. Castleden I. R. Tanz S. K. Aryamanesh N. Millar A. H. (2017). SUBA4: the interactive data analysis Centre for Arabidopsis subcellular protein locations. Nucleic Acids Res. 45, D1064–D1074. doi: 10.1093/nar/gkw1041, PMID: 27899614 PMC5210537

[ref26] Hunter J. D. (2007). Matplotlib: a 2D graphics environment. Comput. Sci. Eng. 9, 90–95. doi: 10.1109/MCSE.2007.55

[ref27] Jiang J. Gai Z. Wang Y. Fan K. Sun L. Wang H.. (2018). Comprehensive proteome analyses of lysine acetylation in tea leaves by sensing nitrogen nutrition. BMC Genomics 19:840. doi: 10.1186/s12864-018-5250-4, PMID: 30477445 PMC6258439

[ref28] Jones P. Binns D. Chang H.-Y. Fraser M. Li W. McAnulla C.. (2014). InterProScan 5: genome-scale protein function classification. Bioinformatics 30, 1236–1240. doi: 10.1093/bioinformatics/btu031, PMID: 24451626 PMC3998142

[ref29] Julca I. Ferrari C. Flores-Tornero M. Proost S. Lindner A.-C. Hackenberg D.. (2021). Comparative transcriptomic analysis reveals conserved programmes underpinning organogenesis and reproduction in land plants. Nat. Plants 7, 1143–1159. doi: 10.1038/s41477-021-00958-2, PMID: 34253868

[ref30] Kang D. Ahn H. Lee S. Lee C.-J. Hur J. Jung W.. (2019). StressGenePred: a twin prediction model architecture for classifying the stress types of samples and discovering stress-related genes in arabidopsis. BMC Genomics 20:949. doi: 10.1186/s12864-019-6283-z, PMID: 31856731 PMC6923958

[ref31] Kim H. Smith H. B. Mathis C. Raymond J. Walker S. I. (2019). Universal scaling across biochemical networks on earth. Sci. Adv. 5:eaau0149. doi: 10.1126/sciadv.aau0149, PMID: 30746442 PMC6357746

[ref32] Kim S. C. Sprung R. Chen Y. Xu Y. Ball H. Pei J.. (2006). Substrate and functional diversity of lysine acetylation revealed by a proteomics survey. Mol. Cell 23, 607–618. doi: 10.1016/j.molcel.2006.06.026, PMID: 16916647

[ref33] Klopfenstein D. V. Zhang L. Pedersen B. S. Ramírez F. Warwick Vesztrocy A. Naldi A.. (2018). GOATOOLS: a python library for gene ontology analyses. Sci. Rep. 8:10872. doi: 10.1038/s41598-018-28948-z, PMID: 30022098 PMC6052049

[ref34] Kozlowski L. P. (2016). IPC–Isoelectric Point Calculator. Biol. Direct 11:55. doi: 10.1186/s13062-016-0159-9, PMID: 27769290 PMC5075173

[ref35] Kulmanov M. Khan M. A. Hoehndorf R. (2018). DeepGO: predicting protein functions from sequence and interactions using a deep ontology-aware classifier. Bioinformatics 34, 660–668. doi: 10.1093/bioinformatics/btx624, PMID: 29028931 PMC5860606

[ref36] Kurotani A. Tokmakov A. A. Kuroda Y. Fukami Y. Shinozaki K. Sakurai T. (2014). Correlations between predicted protein disorder and post-translational modifications in plants. Bioinformatics 30, 1095–1103. doi: 10.1093/bioinformatics/btt762, PMID: 24403539 PMC3982157

[ref37] Lan Y. Sun R. Ouyang J. Ding W. Kim M.-J. Wu J.. (2021). AtMAD: *Arabidopsis thaliana* multi-omics association database. Nucleic Acids Res. 49, D1445–D1451. doi: 10.1093/nar/gkaa1042, PMID: 33219693 PMC7778929

[ref38] Lee T. Yang S. Kim E. Ko Y. Hwang S. Shin J.. (2015). AraNet v2: an improved database of co-functional gene networks for the study of *Arabidopsis thaliana* and 27 other nonmodel plant species. Nucleic Acids Res. 43, D996–D1002. doi: 10.1093/nar/gku1053, PMID: 25355510 PMC4383895

[ref39] Lemaître G. Nogueira F. Aridas C. K. (2017). Imbalanced-learn: a python toolbox to tackle the curse of imbalanced datasets in machine learning. J. Mach. Learn. Res. 18, 1–5. doi: 10.48550/arXiv.1609.06570

[ref40] Li G. Zheng B. Zhao W. Ren T. Zhang X. Ning T.. (2021). Global analysis of lysine acetylation in soybean leaves. Sci. Rep. 11:17858. doi: 10.1038/s41598-021-97338-9, PMID: 34504199 PMC8429545

[ref41] Libbrecht M. W. Noble W. S. (2015). Machine learning applications in genetics and genomics. Nat. Rev. Genet. 16, 321–332. doi: 10.1038/nrg3920, PMID: 25948244 PMC5204302

[ref42] Littmann M. Heinzinger M. Dallago C. Olenyi T. Rost B. (2021). Embeddings from deep learning transfer GO annotations beyond homology. Sci. Rep. 11:1160. doi: 10.1038/s41598-020-80786-0, PMID: 33441905 PMC7806674

[ref43] Lloyd J. P. Seddon A. E. Moghe G. D. Simenc M. C. Shiu S.-H. (2015). Characteristics of plant essential genes allow for within- and between-species prediction of lethal mutant phenotypes. Plant Cell 27, 2133–2147. doi: 10.1105/tpc.15.00051, PMID: 26286535 PMC4568498

[ref44] Mahood E. H. Kruse L. H. Moghe G. D. (2020). Machine learning: a powerful tool for gene function prediction in plants. Appl. Plant Sci. 8:e11376. doi: 10.1002/aps3.11376, PMID: 32765975 PMC7394712

[ref45] Moore B. M. Wang P. Fan P. Leong B. Schenck C. A. Lloyd J. P.. (2019). Robust predictions of specialized metabolism genes through machine learning. Proc. Natl. Acad. Sci. 116, 2344–2353. doi: 10.1073/pnas.1817074116, PMID: 30674669 PMC6369796

[ref46] Mustafin Z. S. Zamyatin V. I. Konstantinov D. K. Doroshkov A. V. Lashin S. A. Afonnikov D. A. (2019). Phylostratigraphic analysis shows the earliest origination of the abiotic stress associated genes in *A. thaliana*. Gene 10:963. doi: 10.3390/genes10120963, PMID: 31766757 PMC6947294

[ref47] Mutwil M. Usadel B. Schutte M. Loraine A. Ebenhoh O. Persson S. (2010). Assembly of an interactive correlation network for the Arabidopsis genome using a novel heuristic clustering algorithm. Plant Physiol. 152, 29–43. doi: 10.1104/pp.109.145318, PMID: 19889879 PMC2799344

[ref48] Narasumani M. Harrison P. M. (2018). Discerning evolutionary trends in post-translational modification and the effect of intrinsic disorder: analysis of methylation, acetylation and ubiquitination sites in human proteins. PLoS Comput. Biol. 14:e1006349. doi: 10.1371/journal.pcbi.1006349, PMID: 30096183 PMC6105011

[ref49] Ng J. W. X. Tan Q. W. Ferrari C. Mutwil M. (2020). Diurnal.Plant.Tools: comparative transcriptomic and co-expression analyses of diurnal gene expression of the archaeplastida kingdom. Plant Cell Physiol. 61, 212–220. doi: 10.1093/pcp/pcz176, PMID: 31501868

[ref50] Obayashi T. Hayashi S. Saeki M. Ohta H. Kinoshita K. (2009). ATTED-II provides coexpressed gene networks for Arabidopsis. Nucleic Acids Res. 37, D987–D991. doi: 10.1093/nar/gkn807, PMID: 18953027 PMC2686564

[ref51] Panni S. Lovering R. C. Porras P. Orchard S. (2020). Non-coding RNA regulatory networks. Biochim. Biophys. Acta BBA-Gene Regul. Mech. 1863:194417. doi: 10.1016/j.bbagrm.2019.194417 31493559

[ref52] Pastor-Satorras R. Smith E. Solé R. V. (2003). Evolving protein interaction networks through gene duplication. J. Theor. Biol. 222, 199–210. doi: 10.1016/S0022-5193(03)00028-6, PMID: 12727455

[ref53] Pedregosa F. Varoquaux G. Gramfort A. Michel V. Thirion B. Grisel O.. (2011). Scikit-learn: machine learning in python. J. Mach. Learn. Res. 12, 2825–2830. doi: 10.48550/arXiv.1201.049

[ref54] Pérez-Clemente R. M. Vives V. Zandalinas S. I. López-Climent M. F. Muñoz V. Gómez-Cadenas A. (2013). Biotechnological approaches to study plant responses to stress. Biomed. Res. Int. 2013:654120. doi: 10.1155/2013/654120, PMID: 23509757 PMC3591138

[ref55] Pimentel H. Bray N. L. Puente S. Melsted P. Pachter L. (2017). Differential analysis of RNA-seq incorporating quantification uncertainty. Nat. Methods 14, 687–690. doi: 10.1038/nmeth.4324, PMID: 28581496

[ref56] Proceedings of the Python in Science Conference (2008). Exploring network structure, dynamics, and function using NetworkX. Available at: http://conference.scipy.org/proceedings/SciPy2008/paper_2/ (Accessed November 26, 2021).

[ref57] Proost S. Mutwil M. (2018). CoNekT: an open-source framework for comparative genomic and transcriptomic network analyses. Nucleic Acids Res. 46, W133–W140. doi: 10.1093/nar/gky336, PMID: 29718322 PMC6030989

[ref58] Rhee S. Y. Mutwil M. (2014). Towards revealing the functions of all genes in plants. Trends Plant Sci. 19, 212–221. doi: 10.1016/j.tplants.2013.10.006, PMID: 24231067

[ref59] Sasaki E. Takahashi C. Asami T. Shimada Y. (2011). AtCAST, a tool for exploring gene expression similarities among DNA microarray experiments using networks. Plant Cell Physiol. 52, 169–180. doi: 10.1093/pcp/pcq185, PMID: 21113043

[ref60] Shannon P. Markiel A. Ozier O. Baliga N. S. Wang J. T. Ramage D.. (2003). Cytoscape: a software environment for integrated models of biomolecular interaction networks. Genome Res. 13, 2498–2504. doi: 10.1101/gr.1239303, PMID: 14597658 PMC403769

[ref61] Stark C. Breitkreutz B.-J. Reguly T. Boucher L. Breitkreutz A. Tyers M. (2006). BioGRID: a general repository for interaction datasets. Nucleic Acids Res. 34, D535–D539. doi: 10.1093/nar/gkj109, PMID: 16381927 PMC1347471

[ref62] Sureyya Rifaioglu A. Doğan T. Jesus Martin M. Cetin-Atalay R. Atalay V. (2019). DEEPred: automated protein function prediction with multi-task feed-forward deep neural networks. Sci. Rep. 9:7344. doi: 10.1038/s41598-019-43708-3, PMID: 31089211 PMC6517386

[ref63] Tang M. Li B. Zhou X. Bolt T. Li J. J. Cruz N.. (2021). A genome-scale TF-DNA interaction network of transcriptional regulation of Arabidopsis primary and specialized metabolism. Mol. Syst. Biol. 17:e10625. doi: 10.15252/msb.202110625, PMID: 34816587 PMC8611409

[ref64] Van Dongen S. M. (2000). Graph clustering by flow simulation.

[ref65] van Noort V. Snel B. Huynen M. A. (2004). The yeast coexpression network has a small-world, scale-free architecture and can be explained by a simple model. EMBO Rep. 5, 280–284. doi: 10.1038/sj.embor.7400090, PMID: 14968131 PMC1299002

[ref66] Waskom M. L. (2021). seaborn: statistical data visualization. J. Open Source Softw. 6:3021. doi: 10.21105/joss.03021

[ref67] Willems P. Horne A. Van Parys T. Goormachtig S. De Smet I. Botzki A.. (2019). The plant PTM viewer, a central resource for exploring plant protein modifications. Plant J. 99, 752–762. doi: 10.1111/tpj.14345, PMID: 31004550

[ref68] Xiong Y. Peng X. Cheng Z. Liu W. Wang G.-L. (2016). A comprehensive catalog of the lysine-acetylation targets in rice (*Oryza sativa*) based on proteomic analyses. J. Proteome 138, 20–29. doi: 10.1016/j.jprot.2016.01.019, PMID: 26836501

[ref69] Yilmaz A. Mejia-Guerra M. K. Kurz K. Liang X. Welch L. Grotewold E. (2011). AGRIS: the Arabidopsis gene regulatory information server, an update. Nucleic Acids Res. 39, D1118–D1122. doi: 10.1093/nar/gkq1120, PMID: 21059685 PMC3013708

[ref70] Zaborowski A. B. Walther D. (2020). Determinants of correlated expression of transcription factors and their target genes. Nucleic Acids Res. 48, 11347–11369. doi: 10.1093/nar/gkaa927, PMID: 33104784 PMC7672440

[ref71] Zhai J. Tang Y. Yuan H. Wang L. Shang H. Ma C. (2016). A meta-analysis based method for prioritizing candidate genes involved in a pre-specific function. Front. Plant Sci. 7:1914. doi: 10.3389/fpls.2016.01914, PMID: 28018423 PMC5156684

[ref72] Zhang Y. Song L. Liang W. Mu P. Wang S. Lin Q. (2016). Comprehensive profiling of lysine acetylproteome analysis reveals diverse functions of lysine acetylation in common wheat. Sci. Rep. 6:21069. doi: 10.1038/srep21069, PMID: 26875666 PMC4753473

[ref73] Zwaenepoel A. Diels T. Amar D. Van Parys T. Shamir R. Van de Peer Y.. (2018). Morph DB: prioritizing genes for specialized metabolism pathways and gene ontology categories in plants. Front. Plant Sci. 9:352. doi: 10.3389/fpls.2018.00352, PMID: 29616063 PMC5867296

