# Supplementary material for: Feature importance network reveals novel functional relationships between biological features in Arabidopsis thaliana
Source: Front Plant Sci. 2022 Sep 23;13:944992. doi: 10.3389/fpls.2022.944992 (PMC9539877; doi:10.3389/fpls.2022.944992)
Supplement: SUPPLEMENTARY DATA 1 — Machine learning dataset, zip file. [file Data_Sheet_1.zip › Supplementary Figures.PDF]

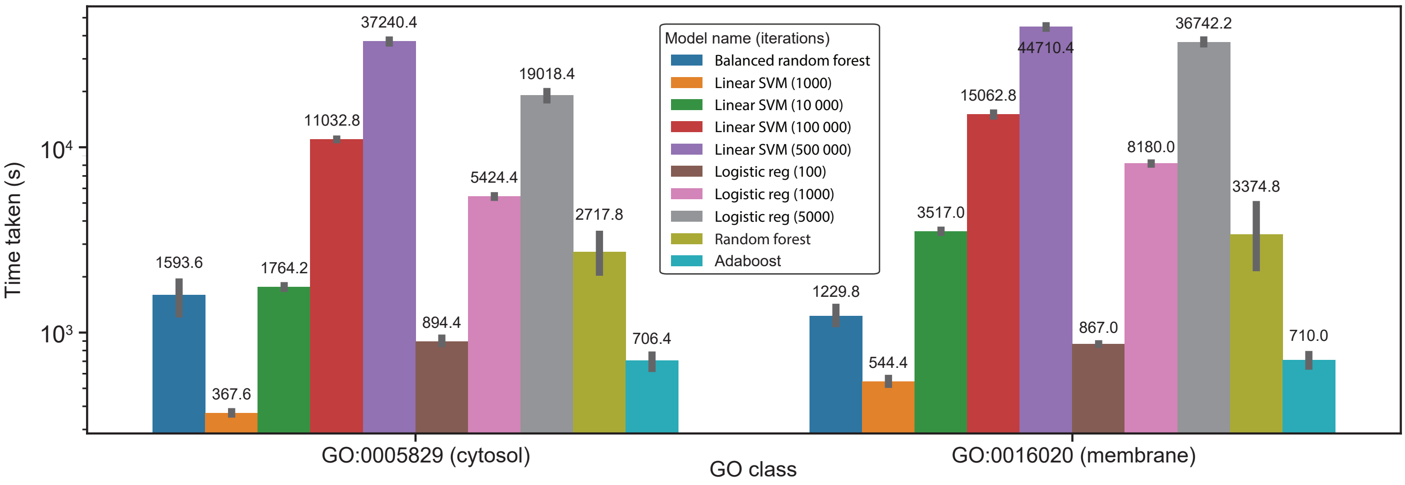

Figure S1. Shows time taken for each individual GO class. Time trial repeat - running all machine learning models on 2 GO classes, 5 repeats per model, to allow for all models to be compared on a common basis.

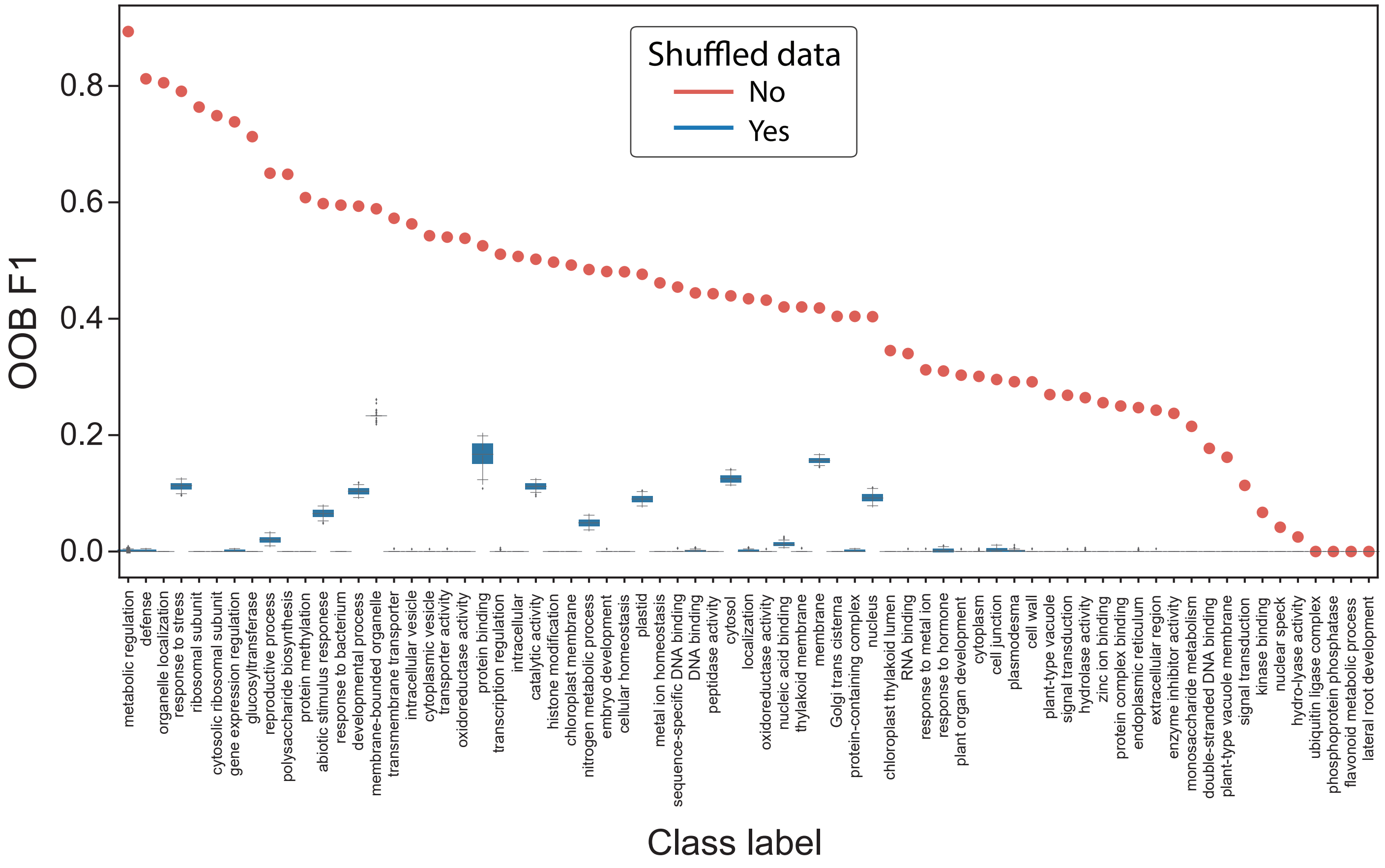

Figure S2. Random forest OOB scores when trained on shuffled data (blue) or actual data (orange). Some GO term descriptions have been summarised to save space, full descriptions are given in Table S9.

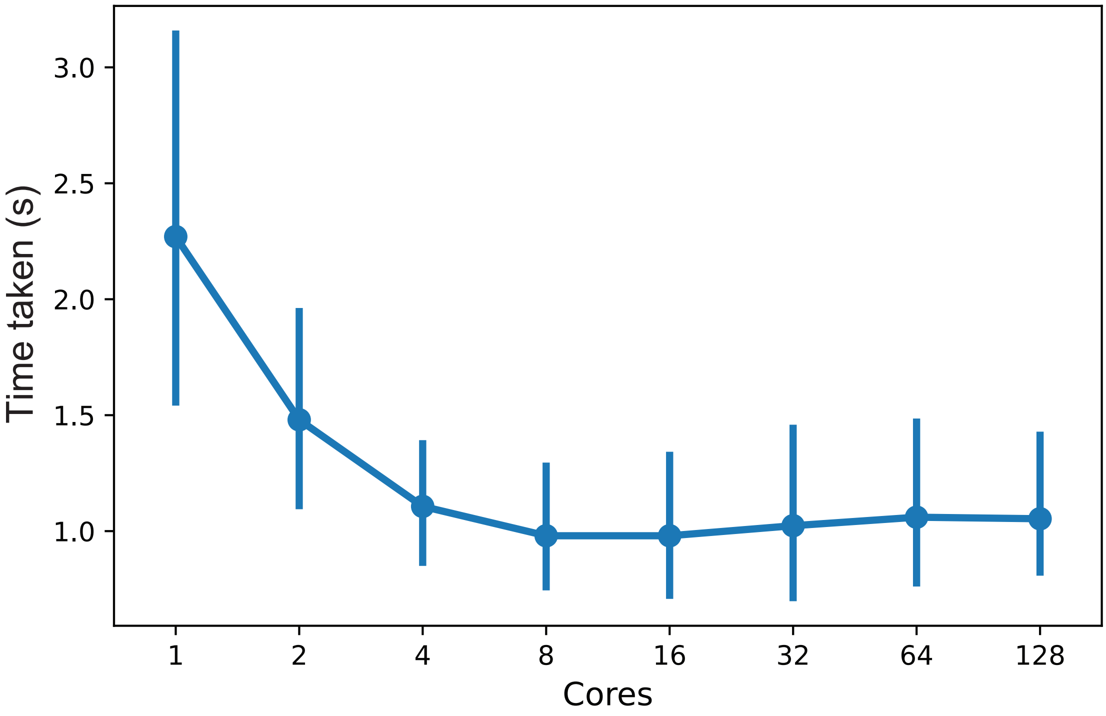

Figure S3. Line plot showing time taken to train random forest models on 5 GO classes, GO:0043229 (intracellular organelle), GO:0005774 (vacuolar membrane), GO:0048827 (phyllome development), GO:0030001 (metal ion transport) and GO:0016836 (hydro-lyase activity).

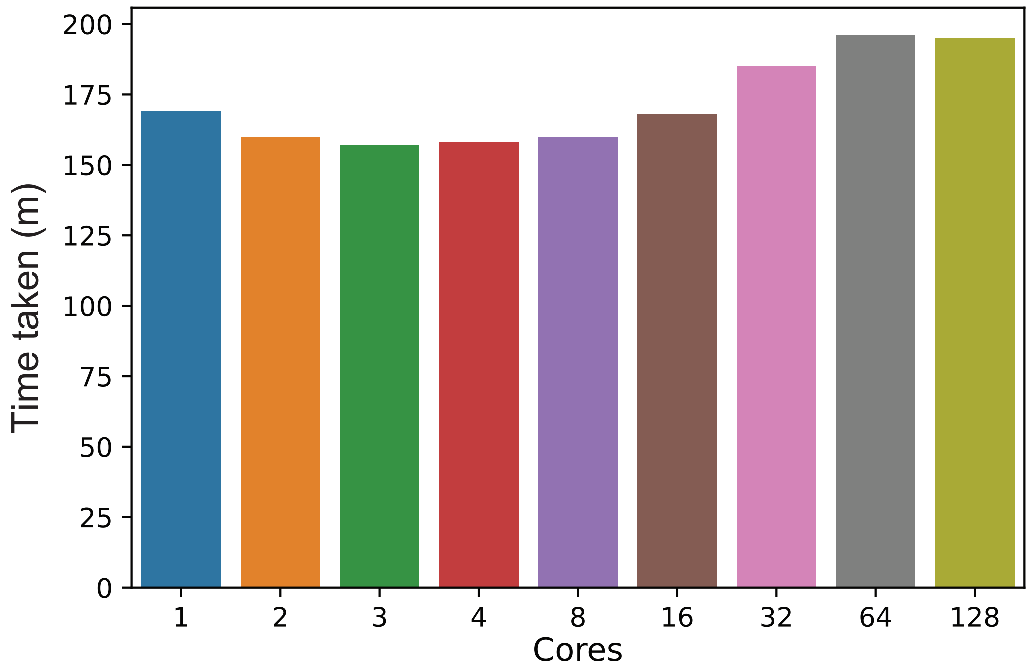

Figure S4. Bar graph showing time taken to train random forest models on all GO classes.

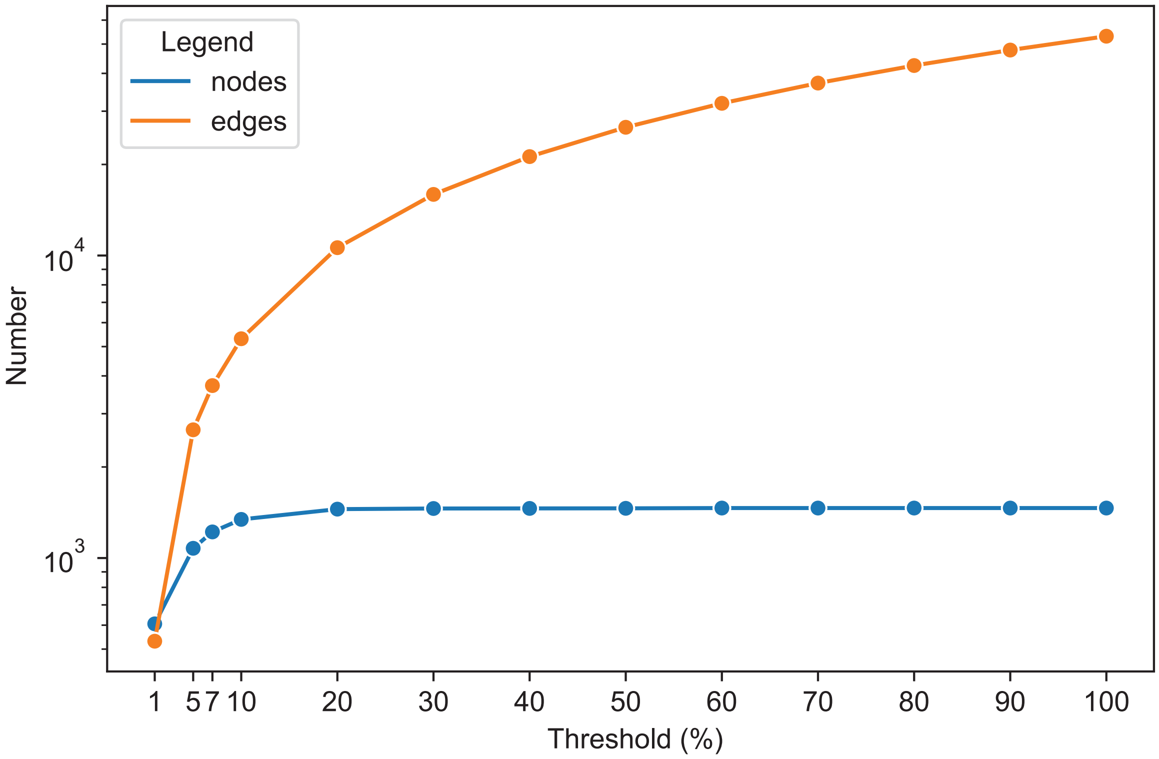

Figure S5. Line plot showing the number of nodes and edges in the feature importance network (FIN) after applying different mutual rank thresholds. These thresholds are the top X% of mutual ranks (feature pairs, otherwise known as edges) selected from the FIN.
